# Adrenergic receptor agonists induce the differentiation of pluripotent stem cell-derived hepatoblasts into hepatocyte-like cells

**DOI:** 10.1038/s41598-017-16858-5

**Published:** 2017-12-01

**Authors:** Maki Kotaka, Taro Toyoda, Katsutaro Yasuda, Yuko Kitano, Chihiro Okada, Akira Ohta, Akira Watanabe, Motonari Uesugi, Kenji Osafune

**Affiliations:** 1 Center for iPS Cell Research and Application (CiRA), Kyoto University, 53 Kawahara-cho, Shogoin, Sakyo-ku, Kyoto, 606-8507 Japan; 2 Institute for Integrated Cell-Material Sciences (iCeMS), Kyoto University, 53 Kawahara-cho, Shogoin, Sakyo-ku, Kyoto, 606-8507 Japan; 3 Mitsubishi Space Software Co., Ltd., 5-4-36 Tsukaguchi-honmachi, Amagasaki, Hyogo, 661-0001 Japan; 4 Institute for Chemical Research, Kyoto University, Gokasho Uji-city, Kyoto, 611-0011 Japan

## Abstract

Current induction methods of hepatocytes from human induced pluripotent stem cells (hiPSCs) are neither low cost nor stable. By screening a chemical library of 1,120 bioactive compounds and known drugs, we identified the α1-adrenergic receptor agonist methoxamine hydrochloride as a small molecule that promotes the differentiation of hiPSC-derived hepatoblasts into ALBUMIN^+^ hepatocyte-like cells. Other α1-adrenergic receptor agonists also induced the differentiation of hepatocyte-like cells, and an α1-receptor antagonist blocked the hepatic-inducing activity of methoxamine hydrochloride and that of the combination of hepatocyte growth factor (HGF) and Oncostatin M (OsM), two growth factors often used for the induction of hepatoblasts into hepatocyte-like cells. We also confirmed that treatment with methoxamine hydrochloride activates the signal transducer and activator of transcription 3 (STAT3) pathway downstream of IL-6 family cytokines including OsM. These findings allowed us to establish hepatic differentiation protocols for both mouse embryonic stem cells (mESCs) and hiPSCs using small molecules at the step from hepatoblasts into hepatocyte-like cells. The results of the present study suggest that α1-adrenergic agonists induce hepatocyte-like cells by working downstream of HGF and OsM to activate STAT3.

## Introduction

Orthotopic liver transplantation is the only radical treatment for chronic liver diseases, but the majority of patients die due to the shortage of donor livers^1^. Hepatocyte transplantation has recently become a treatment of acute liver failure and life-threatening metabolic liver diseases^2^. However, this strategy is also hampered by the shortage of donor hepatocyte sources. Although cryopreserved primary human hepatocytes are useful in liver cell transplantation and drug screening, they rapidly lose their functions and hardly proliferate in *in vitro* culture systems.

Human embryonic stem cells (hESCs) and induced pluripotent stem cells (hiPSCs) are an attractive alternative cell source for primary human hepatocytes due to their ability to unlimitedly self-renew and to differentiate into any cell types of the body, including hepatocytes^3–5^. Stepwise differentiation methods to generate hepatic lineage cells from hESCs/hiPSCs have been developed that mimic the developmental process of liver^6–12^. In these protocols, definitive endoderm cells are initially induced by treatment with a high concentration of activin A, followed by hepatoblast and hepatocyte differentiation using growth factors, such as hepatocyte growth factor (HGF) and Oncostatin M (OsM). Although combination treatment with these two factors has usually been used for the induction of hepatic lineage, the downstream signals of the factors remain to be elucidated.

Knowing these signals is important, because growth factors are expensive and show large lot-to-lot variability, which limits their practical and clinical use. On the other hand, small-molecule inducers are more cost-effective, easier to handle, and possibly more efficient than growth factors at directed differentiation^13^. Screening for chemical compounds in an unbiased manner has been used to identify novel small molecules that induce the differentiation of mouse ESCs (mESCs) into definitive endoderm^14^ and pancreatic endocrine cells^15^ and the differentiation of hESCs and/or hiPSCs into intermediate mesoderm^16^, hepatocytes^13,17^, pancreatic progenitors^18^ and cardiomyocytes^19^.

Adrenergic receptors are expressed in many cell types and are the targets of catecholamines, such as noradrenaline (norepinephrine) and adrenaline (epinephrine)^20^. These receptors are largely classified into two types, α and β, with subtypes α1, α2, β1, β2 and β3. Signals through adrenergic receptors are involved in numerous biological functions, such as the activation of sympathetic nervous systems, smooth muscle contraction and relaxation, glycogenolysis and gluconeogenesis, and increased cardiac output. Regarding liver, noradrenaline or a β-adrenergic receptor agonist isoproterenol has been related to the DNA synthesis in adult rat hepatocytes^21–24^. It has also been reported that fetal rat hepatocytes in culture under proliferative conditions, namely, in the presence of epidermal growth factor (EGF), respond to glucagon and noradrenaline to increase Albumin mRNA and protein expression levels^25^. However, there have been no reports so far describing the signals through which adrenergic receptors may regulate the differentiation of hepatic lineage cells from pluripotent stem cells (PSCs).

In this study, we screened a chemical library that consists of 1,120 compounds in order to identify small molecules that can induce hiPSC-derived hepatoblasts into ALBUMIN^+^ hepatocyte-like cells in the absence of HGF and OsM. We identified one hit compound, methoxamine hydrochloride, which is an α1-adrenergic receptor agonist, and used it to establish differentiation protocols from both mESCs and hiPSCs into hepatocyte-like cells. We also found that other α1-adrenergic receptor agonists can induce hiPSC-derived hepatoblasts into hepatocyte-like cells without HGF and OsM, and that the addition of an α1-adrenergic receptor antagonist to hepatic differentiation cultures using HGF and OsM abolished the hepatic inducing activity. This report is the first to show that adrenergic receptor agonists act as inducers for hepatic lineage differentiation from pluripotent stem cells and to suggest adrenergic receptor signals may be downstream of HGF and OsM in hepatic differentiation.

## Results

### Chemical screening identified the α1-adrenergic receptor agonist methoxamine hydrochloride as a hepatic inducer

**Figure 1:**
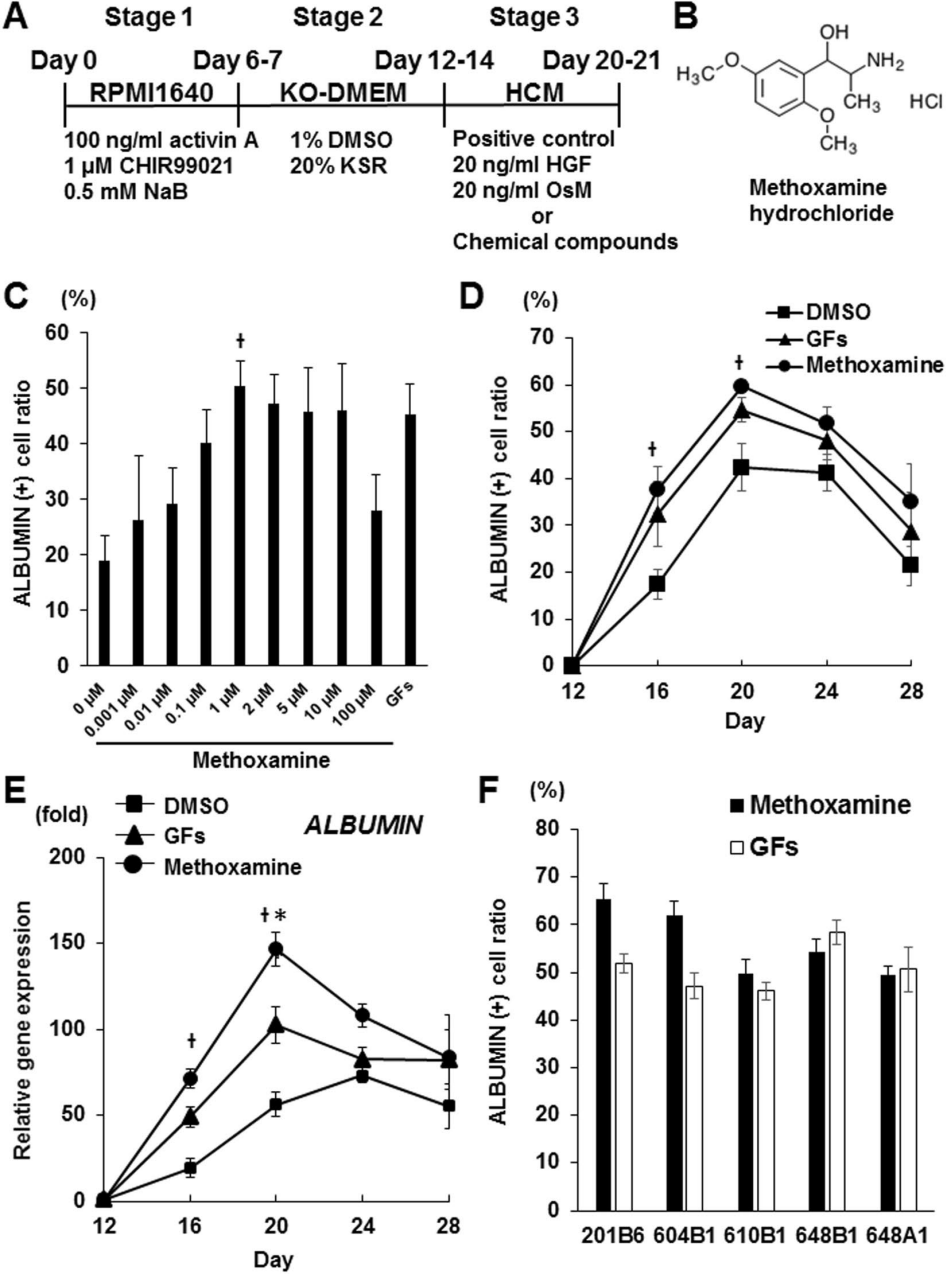
Methoxamine treatment induces hiPSC-derived hepatoblasts into ALBUMIN^+^ cells. (**A**) A schematic drawing of the screening procedure. (**B**) The chemical structure of methoxamine hydrochloride. (**C**) Dose-dependent induction of ALBUMIN^+^ cells by methoxamine treatment. P < 0.05 (One-way analysis of variance (ANOVA) with Bonferroni’s Multiple Comparison Test). (D, E) Time course analyses of effects of methoxamine treatment on the induction rate of ALBUMIN^+^ cells (**D**) and the *ALBUMIN* expression (**E**). P < 0.05 vs DMSO and *P < 0.05 vs GFs (One-way analysis of variance (ANOVA) with Bonferroni’s Multiple Comparison Test). (**F**) Hepatic differentiation of multiple hiPSC lines using methoxamine treatment or GFs as analyzed by the induction rate of ALBUMIN^+^ cells on day 20 (Stage 3, day 8). The data are presented as mean ± SE from three independent experiments (n = 3) in (**C**,**F**). NaB: sodium butyrate, KO-DMEM: knockout-DMEM, KSR: knockout serum replacement, HCM: hepatocyte culture medium, HGF: hepatocyte growth factor, OsM: oncostatin M, GFs: HGF+OsM.

In order to identify small molecules that can efficiently induce ALBUMIN^+^ cells, we designed a chemical screening strategy (Fig. 1A). We generated hepatic lineage cells from a fibroblast-derived hiPSC line, 201B6^3^, by using a modified version of a previously reported differentiation protocol^11^. We initiated the screening with hiPSC-derived hepatoblasts on culture day 14 and screened the Prestwick Chemical library, which consists of 1,120 bioactive compounds and known drugs, in 384-well plates. Positive hits were defined as compounds that induced hiPSC-derived hepatoblasts into ALBUMIN^+^ cells at efficiencies equal to or better than the control stimulus (HGF and OsM). Out of the 1,120 compounds examined, we found one hit compound, methoxamine hydrochloride (hereafter called methoxamine), which is an α1-adrenergic receptor agonist (Fig. 1B). The induction rate of ALBUMIN^+^ cells in the populations treated with methoxamine (33.7%; n = 2) was similar to that of the control stimulus (31.3%; n = 2).

We then examined various concentrations and treatment durations, as analyzed by anti-ALBUMIN immunocytochemistry in order to determine the optimal induction conditions of ALBUMIN^+^ cells by methoxamine. We found that the induction efficiency of ALBUMIN^+^ cells varied with the size of the cell culture plates. We therefore used 24-well plates, which showed higher differentiation efficiency than 384-well plates, for the following analysis. The results of titrating methoxamine from 0.001 μM to 100 μM showed that the inducing activity is dose dependent and highest efficiency is at 1 μM (P < 0.05; Fig. 1C). The temporal pattern of the induction rate of ALBUMIN^+^ cells showed a gradual increase with a peak on culture day 20 after 8 days of methoxamine treatment (Fig. 1D), which is consistent with the result of the mRNA expression of *ALBUMIN* by qRT-PCR, showing a significantly higher expression level by methoxamine treatment than by treatment with HGF and OsM on day 20 (P < 0.05; Fig. 1E).

hESCs/hiPSCs differ in differentiation potential among cell lines^11,26^. We thus examined our methoxamine-based induction protocol for ALBUMIN^+^ cells using multiple hiPSC lines. The protocol was effective on 201B6, peripheral blood-derived hiPSC lines (604B1, 648A1 and 648B1) and a cord blood-derived hiPSC line (610B1) (Fig. 1F)^3,11,27^. These results indicate that our induction protocol using methoxamine for ALBUMIN^+^ cells has broad application.

### Characterization of hepatic cells induced with methoxamine treatment

**Figure 2:**
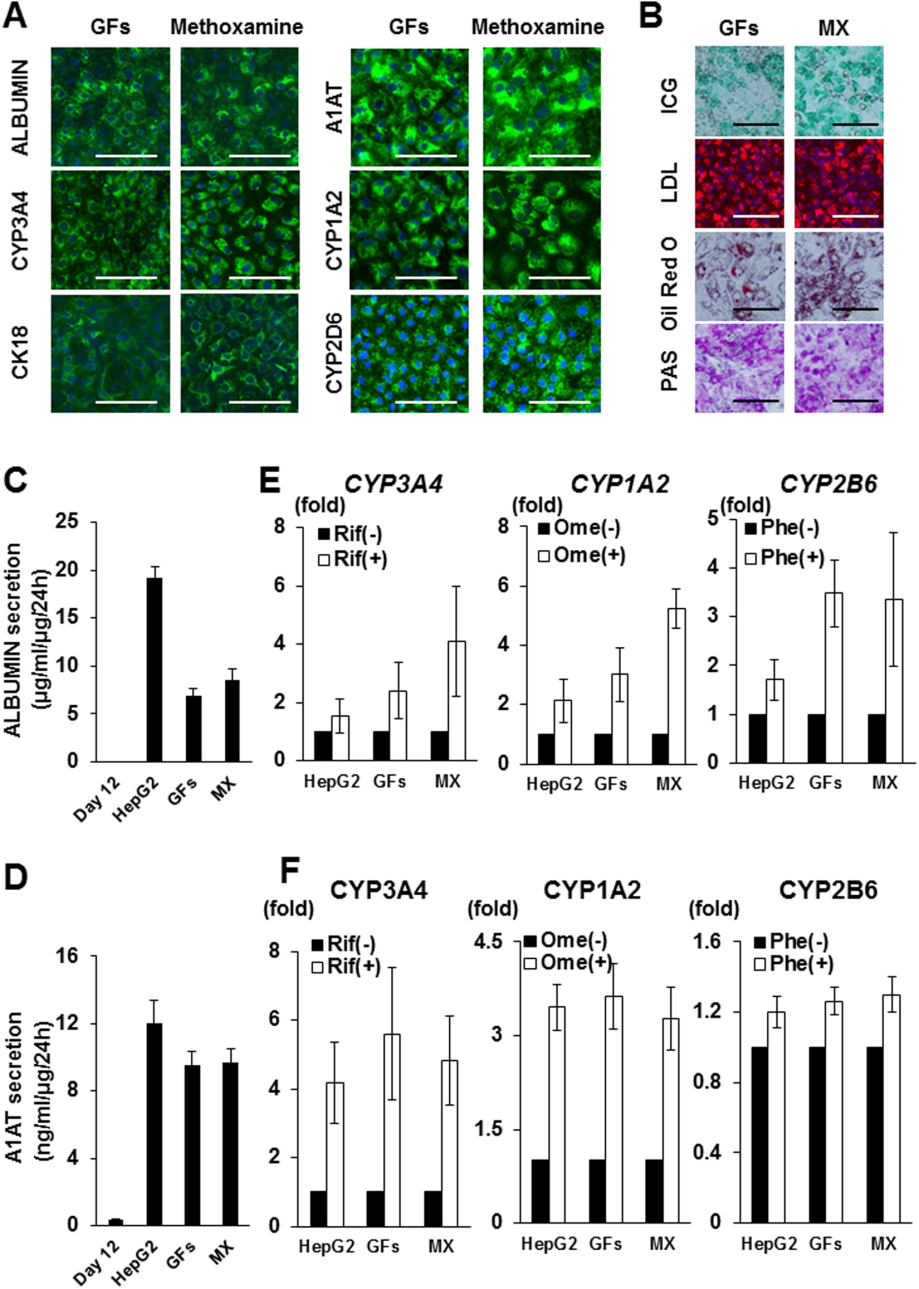
Functional analysis of hepatic cells induced with methoxamine treatment. (**A**) Immunostaining analyses of hepatic markers on day 20 cells induced with HGF and OsM (GFs) or methoxamine. (**B**) The uptake of indocyanine green (ICG; top panels) and low-density lipoprotein (LDL; middle upper panels), oil Red O staining (middle lower panels) and Periodic acid-Schiff staining (bottom panels) were examined in day 20 cells induced with GFs or methoxamine (MX). (**C**,**D**) ELISA analyses of ALBUMIN (**C**) and α1-ANTITRYPSIN (A1AT; D) secretion by hiPSC-derived differentiated cells on culture days 12 (Stage 2, day 6) and 20 (Stage 3, day 8) after treatment with GFs or MX. HepG2 cells were used as control. Values were normalized to total protein amounts. (**E**,**F**) Induction of *CYP3A4*, *CYP1A2* and *CYP2B6* mRNAs (**E**) and CYP3A4, CYP1A2 and CYP2B6 enzymes (**F**) by 48 hour treatment with 40 μM rifampicin (Rif), 40 μM omeprazole (Ome) and 100 μM phenobarbital (Phe), respectively, in day 20 cells induced with GFs or MX. HepG2 cells were used as control. Values were normalized to those of samples without drug treatment. The data are presented as mean ± SE from three independent experiments (n = 3) in (**C** **–F**). Scale bars, 100 μm.

In order to confirm that our differentiation protocol using methoxamine generates hepatic lineage cells via the correct developmental pathway, we characterized the cells at each differentiation step induced from two hiPSC lines (201B6 and 585A1) and one hESC line (KhES3) that all expressed the pluripotency markers, OCT4, SOX2 and NANOG (Figs S1 and S2). We confirmed that the three cell lines were differentiated into definitive endoderm and hepatoblasts on days 6 and 12, respectively, by qRT-PCR analyses of the expression of the definitive endoderm markers, *SOX17*, *HHEX*, *FOXA2* and *CER1*, on day 6 (Fig. S3) and the hepatoblast markers, *AFP*, *CEBPα*, *CEBPβ*, *TBX3*, *PROX1* and *GATA4*, on day 12 (Fig. S4). We also found typical hepatocyte morphology of cuboidal shapes (Fig. S5) and the gene expression of various hepatocyte markers (*α1-ANTITRYPSIN* (*A1AT*), *ALBUMIN*, *HNF4A*, *TAT*, *TDO2*, *TTR*, *APOA2*, *ASGR1*, *GSTP1*, *CYP1A1*, *CYP2A6*, *CYP2C19*, *CYP3A4*, *CYP3A5*, *CYP3A7* and *HSP47*) in day 20 cells induced with methoxamine or HGF and OsM (Fig. S6). Immunofluorescence analyses confirmed that the day 20 cells induced with methoxamine treatment were positively stained with the hepatocyte markers, ALBUMIN, CYP3A4, CYTOKERATIN (CK)18, A1AT, CYP1A2, and CYP2D6 (Fig. 2A). We next examined the functionality of ALBUMIN^+^ cells on day 20 induced with methoxamine and confirmed the uptake of indocyanine green (ICG) and low-density lipoprotein (LDL) and cytoplasmic lipid and glycogen storage, as analysed by oil Red O and Periodic acid-Schiff staining, respectively (Fig. 2B). We also confirmed that ALBUMIN and A1AT secretion into the culture media of methoxamine-induced cells, as analyzed by enzyme-linked immunosorbent assay (ELISA), was comparable to that of hepatic cells induced with HGF and OsM (Figs 2C and D). We then examined by qRT-PCR analyses whether methoxamine-treated cells expressed inducible CYP variants after drug treatments and found a 4.1-fold increase in *CYP3A4* by rifampicin, a 5.2-fold increase in *CYP1A2* by omeprazole and a 3.3-fold increase in *CYP2B6* by phenobarbital (Fig. 2E). Furthermore, we performed lytic assay to measure the induction of CYP enzymes and found the induction of CYP3A4 by rifampicin, CYP1A2 by omeprazole and CYP2B6 by phenobarbital in methoxamine-treated cells was similar to that in cells treated with HGF and OsM, although the induction of CYP2B6 was smaller than that of CYP3A4 and CYP1A2 (Fig. 2F). These results suggest that methoxamine treatment can induce hepatocyte-like cells with similar functionality to those induced with HGF and OsM.

### Differentiation of hepatocyte-like cells from mESCs by methoxamine treatment

**Figure 3:**
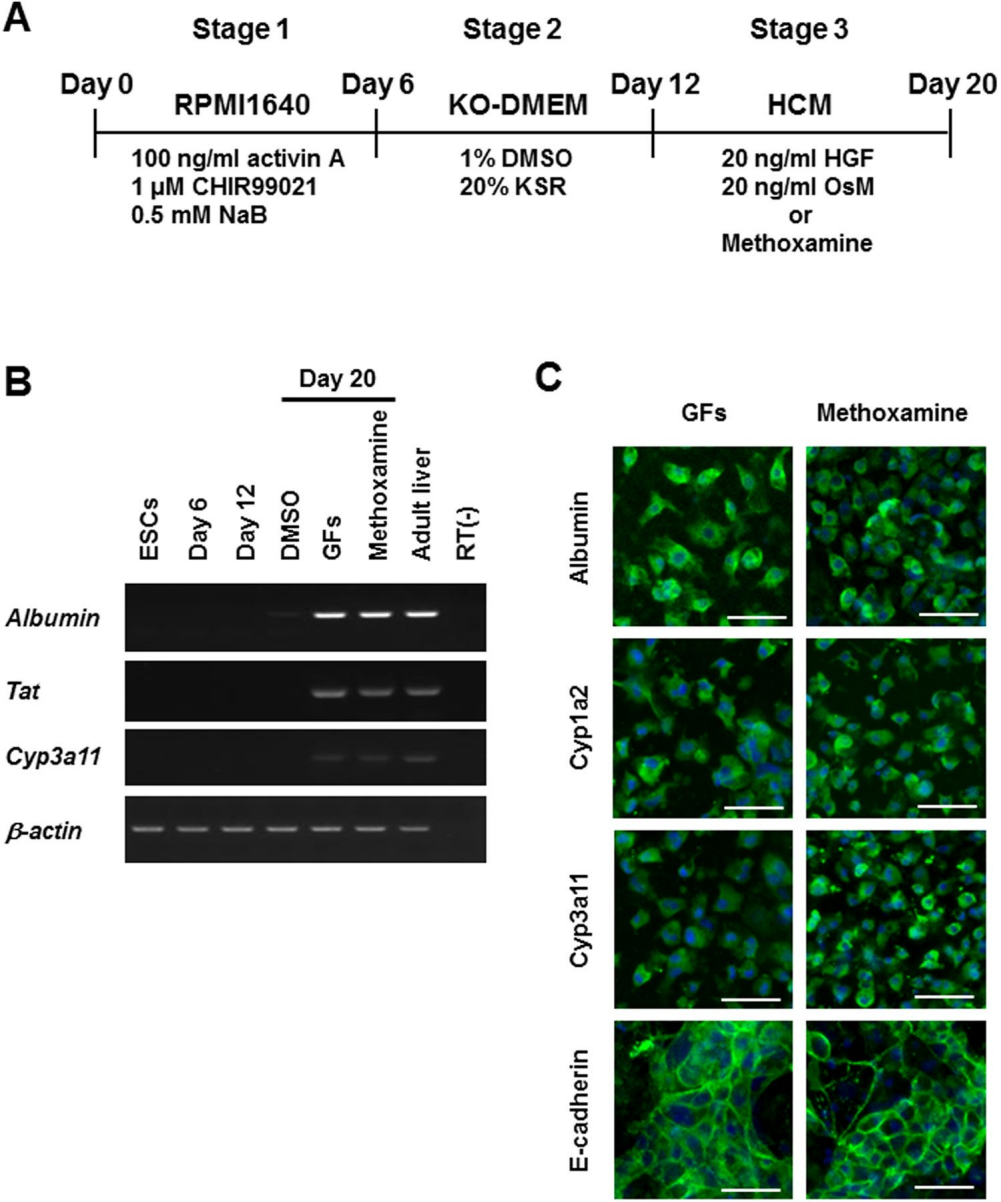
Differentiation of hepatocyte-like cells from mESCs by methoxamine treatment. (**A**) A schematic drawing of the differentiation procedure. (**B**) RT-PCR analyses of the expression of the hepatic marker genes, *Albumin*, *Tat* and *Cyp3a11*, in mESC-derived differentiated cells on days 6 and 12 (Stage 2, day 6) and differentiated cells on day 20 (Stage 3, day 8) after treatment with DMSO, a combination of HGF and OsM (GFs) or methoxamine. Cropped gels are displayed. (**C**) Immunostaining analysis of the protein expression of hepatic markers, Albumin, Cyp1a2, Cyp3a11 and E-cadherin, on the differentiated cells on day 20 after treatment with GFs or methoxamine. Scale bars, 50 μm.

We next examined whether methoxamine treatment can be used for hepatic differentiation from mESCs by modifying our protocol for hiPSCs (Fig. 3A). We found that the expression of hepatic markers, including *Albumin*, *Tat* and *Cyp3a11*, was increased after methoxamine treatment at Stage 3 (Figs 3B and S7). Moreover, the protein expression of hepatic markers, such as Albumin, Cyp1a2, Cyp3a11 and E-cadherin, was also confirmed in the cells treated with methoxamine, as analyzed by immunocytochemistry (Fig. 3C). These results suggest that methoxamine treatment can induce hepatocyte-like cells in the differentiation cultures of both mESCs and hiPSCs.

### Signals through α1-adrenergic receptors induce hepatic differentiation

**Figure 4:**
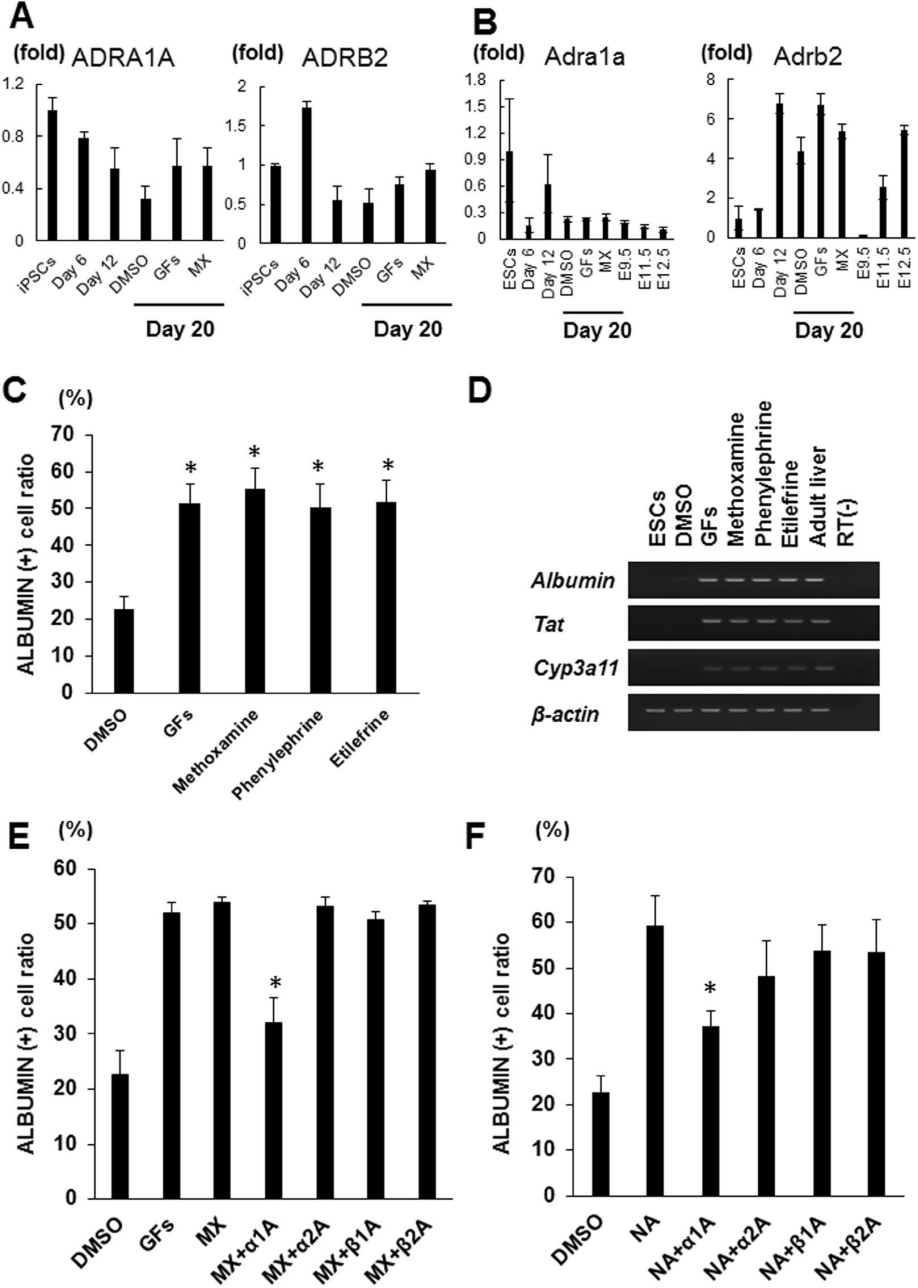
Signals through α1-adrenergic receptors induce hepatic cells.(**A**,**B**) qRT-PCR analyses of the expression of human (**A**) and mouse adrenergic receptor subtypes (**B**) in the cells differentiated from hiPSCs and mESCs, respectively. (**C**) Induction rate of ALBUMIN^+^ cells after 8 days of Stage 3 treatment with three α1-adrenergic receptor agonists, methoxamine, phenylephrine and etilefrine. (**D**) Induction of cells expressing hepatocyte markers from mESCs after 8 days of Stage 3 treatment with methoxamine, phenylephrine or etilefrine. Cropped gels are displayed. (**E**) Effects of adding four adrenergic receptor antagonists, prazosin, yohimbine, metoprolol or butoxamine, on the induction rate of ALBUMIN^+^ cells by methoxamine treatment. (**F**) Effects of adding prazosin, yohimbine, metoprolol or butoxamine on the induction rate of ALBUMIN^+^ cells induced by noradrenaline treatment. The data are presented as mean ± SE from three independent experiments (n = 3) in (**A** **–C**), (**E**) and (**F**). *P < 0.05 (One-way analysis of variance (ANOVA) with Bonferroni’s Multiple Comparison Test) in (**C**), (**E**) and (**F**). GFs: HGF+ OsM, MX: methoxamine, α1 A: α1 receptor antagonist (prazosin), α2 A: α2 receptor antagonist (yohimbine), β1 A: β1 receptor antagonist (metoprolol), β2 A: β2 receptor antagonist (butoxamine).

In an attempt to elucidate the mechanisms of action by which methoxamine induces hepatic cells, we first examined whether adrenergic receptors were expressed in the hepatic differentiation cultures of hiPSCs and mESCs by qRT-PCR analyses. We found that human adrenergic receptors, *ADRA1A* and *ADRB2* (Fig. 4A), and mouse adrenergic receptors, *Adra1a* and *Adrb2* (Fig. 4B), were expressed in undifferentiated states and all three differentiation stages of hiPSCs and mESCs, respectively. Considering the possibility that methoxamine may act through α1-adrenergic receptors to produce hepatocyte-like cells from hepatoblasts, we examined the effects of two other α1-adrenergic receptor agonists, phenylephrine and etilefrine, on the hiPSC-derived hepatoblasts and found they also induced ALBUMIN^+^ cells (Fig. 4C). Furthermore, when used at Stage 3 of mESC differentiation culture, phenylephrine and etilefrine induced cells expressing *Albumin*, *Tat* and *Cyp3a11* (Figs 4D and S8).

We then examined the effects of adding specific adrenergic receptor antagonists, including the α1-adrenergic receptor antagonist prazosin, the α 2-adrenergic receptor antagonist yohimbine, the β1-adrenergic receptor antagonist metoprolol, and the β2-adrenergic receptor antagonist butoxamine to the differentiation cultures treated with methoxamine. As shown in Fig. 4E, only prazosin decreased the induction rate of ALBUMIN^+^ cells, suggesting that signals through α1-adrenergic receptors may be involved in the hepatic differentiation induced by methoxamine.

Noradrenaline is an adrenergic receptor agonist that mainly binds to α1, α2 and β1 receptors^28^. We examined its effects on hepatic differentiation, finding it induced ALBUMIN^+^ cells (Fig. 4F). To determine which adrenergic receptor subtypes were bound, we exposed hiPSC-derived hepatoblasts to the above four adrenergic receptor antagonists in the presence of noradrenaline. Only prazosin decreased the induction rate of ALBUMIN^+^ cells by noradrenaline treatment (Fig. 4F). These results also support the conclusion that signals through α1-adrenergic receptors may induce differentiation into hepatocyte-like cells.

### Generation of ALBUMIN-GFP reporter hiPSC lines

**Figure 5:**
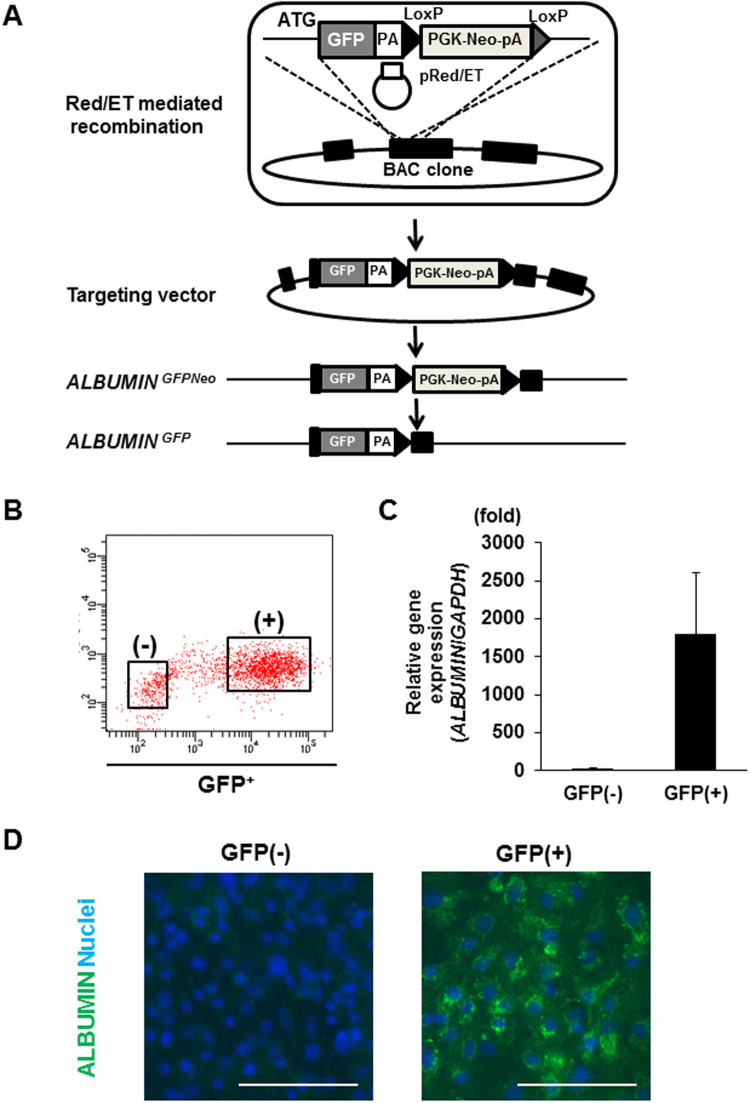
Generation of ALBUMIN-GFP reporter hiPSC lines. (**A**) A schematic representation of the targeting strategy using bacterial artificial chromosome-based vectors to produce ALBUMIN-GFP reporter hiPSC lines. (**B**) The flow cytometric analysis of GFP^+^ cells on day 20. (**C**,**D**) qRT-PCR analysis of *ALBUMIN* expression (**C**) and immunostaining analysis using antibodies against ALBUMIN (**D**) in GFP^−^and GFP^+^ populations isolated on day 20. The data are presented as mean ± SE from three independent experiments (n = 3) in (**C**). Scale bars, 100 μm.

In order to monitor the differentiation of hiPSC-derived hepatocytes, we aimed to establish ALBUMIN-GFP reporter hiPSC lines by adopting a strategy using bacterial artificial chromosome (BAC)-based vectors (Fig. 5A)^29^. 201B6 was transfected with the BAC-based vector, in which the *ALBUMIN*-coding region was replaced with the GFP-Neo cassette. Although we did not find a knockin line with homologous recombination, we obtained transgenic lines. We performed whole genome sequencing of an ALBUMIN-GFP transgenic line, 12C57 C1-1, and the dosage of the sequence read indicated that one copy of the transgene was integrated into the host genome (Fig. S9A). We also identified chimeric sequences that were a consequence of the transgene integration. The breakpoint indicated fusion of the *ALBUMIN*-flanking sequence and chromosome 4 genomic region, but the fusion partner region did not exhibit any genomic features, such as promoter or gene-coding regions. For example, the distance from the breakpoint to the nearest gene *ACO1* was >100 kb, indicating that the effect of transgene integration on gene function is very limited. (Fig. S9B,C). GFP^+^ cells induced from the 12C57 C1-1 cells using the methoxamine-based differentiation protocol were isolated by flow cytometry for qRT-PCR and immunostaining analyses (Fig. 5B). We found that the isolated GFP^+^ cells expressed *ALBUMIN* mRNA (Fig. 5C) and were positively stained with anti-ALBUMIN immunostaining (Fig. 5D). These results indicate that the ALBUMIN-GFP reporter hiPSC line, 12C57 C1-1, can be used to monitor ALBUMIN^+^ cells differentiated from hiPSCs.

### Signals through α1-adrenergic receptors work downstream of HGF and OsM and activate STAT3 pathways

**Figure 6:**
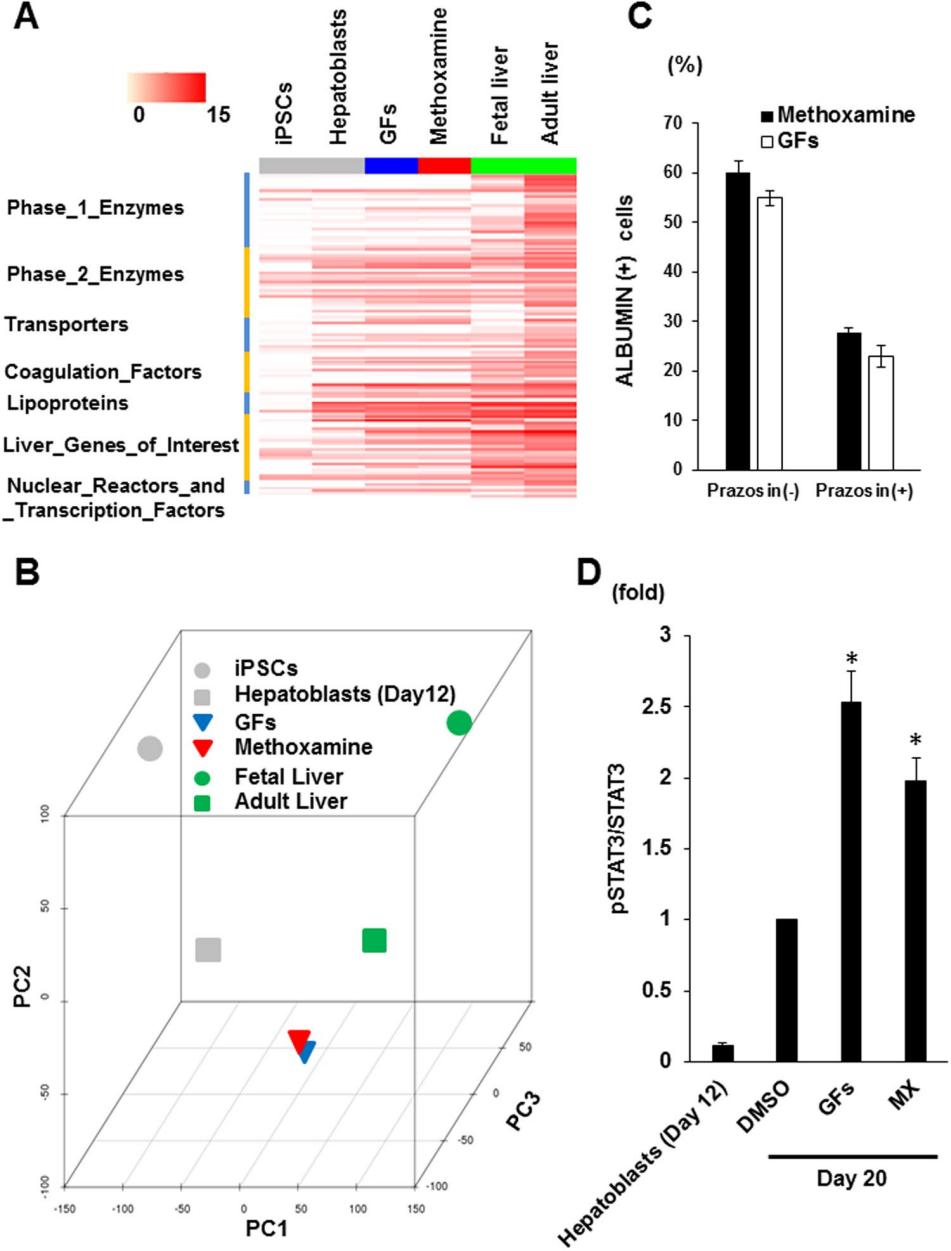
Signals through α1-adrenergic receptors work downstream of HGF and OsM and activate STAT3 pathways.(**A**,**B**) Heat map of gene expressions for selected liver-related transcripts (**A**) and principal component analysis (PCA; **B**) from RNA sequencing data on undifferentiated hiPSCs, hiPSC-derived hepatoblasts, hepatocyte-like cells induced with a combination of HGF and OsM (GFs) or methoxamine, and fetal and adult liver cells. See also Table S1. (**C**) Effects of adding prazosin on the induction rate of ALBUMIN^+^ cells after 8 days of Stage 3 treatment with GFs as well as methoxamine. (**D**) Phosphorylation levels of STAT3 were significantly higher in hiPSC-derived differentiated cells on day 20 (Stage 3, day 8) after treatment with GFs or methoxamine (MX) than those after control DMSO treatment. The relative intensity of phosphorylated STAT3 bands was normalized to those of STAT3. The data from three independent experiments (n = 3) are shown as mean ± SE in (**C**) and (**D**). *P < 0.05 (One-way analysis of variance (ANOVA) with Bonferroni’s Multiple Comparison Test) in (**D**).

Using purified cell populations from the ALBUMIN-GFP reporter hiPSC line, 12C57 C1–1, we compared the global gene expression profiles between hepatocyte-like cells induced with methoxamine and those induced with HGF and OsM by RNA sequencing analysis. A heat map of the gene expressions of liver-related transcripts shows similar gene expression patterns between the two cell groups (Fig. 6A). Furthermore, selected genes in each category were expressed at almost the same level by the two cell populations (Table S1). Principal component analysis (PCA) confirmed that the expression patterns were similar between the two cell groups (Fig. 6B).

Because the two hepatocyte-like cell populations generated with methoxamine or the combination of HGF and OsM showed similar gene expression patterns, we hypothesized that these two treatments use the same signal pathways. We therefore examined the effects of prazosin on the induction of hepatocyte-like cells by HGF and OsM. Interestingly, the addition of prazosin decreased the induction rate of hepatocyte-like cells by HGF and OsM and those by methoxamine (Fig. 6C), suggesting that signals through α1-adrenergic receptors may work downstream of HGF and OsM. It was reported that OsM-induced hepatic differentiation in primary cultures of fetal mouse hepatic cells depends on the signal pathway of signal transducer and activator of transcription 3 (STAT3), a latent transcription factor that is activated by IL-6 family cytokines, including OsM, through their common receptor subunit gp130^30^. The activation of STAT3 pathway was also reported in the treatment of human fetal hepatic progenitor cells with a combination of HGF, OsM and dexamethasone^31^. We thus examined whether a combination of HGF and OsM or methoxamine treatment activates STAT3 in the differentiation from hiPSC-derived hepatoblasts into hepatocyte-like cells by Western blot analysis and found that both treatments significantly increased the phosphorylation levels of STAT3 compared with control DMSO treatment (Figs 6D and S10). These data suggest that α1-adrenergic receptor agonists may induce the differentiation of hepatocyte-like cells by working downstream of HGF and OsM and activating STAT3 pathways.

## Discussion

Human hepatocytes can be used for cell therapy and drug discovery for liver diseases and hepatotoxicity screening of drug compounds^32^. Primary human hepatocytes are often used for these purposes; however, these primary cultured cells easily lose their metabolic functions and stable supply is difficult^33^. While established cell lines, such as HepG2, are readily available, they have low metabolic activity and are not an adequate replacement^34^. Consequently, the absence of human hepatocyte sources has been an obstacle to many medical researches. hESCs/hiPSCs, because of their potential to infinitely proliferate and differentiate into multiple cell types including hepatocytes, are a promising alternative cell source.

Previous works on hepatocyte differentiation from hESCs/hiPSCs have mainly used growth factors as inducers^6–12^. Conventional differentiation methods using growth factors, however, are costly, and the effects of growth factors are unstable between different lots. On the other hand, growth factors can be substituted with low-molecular-weight compounds, which are low cost and highly stable^13,17^. Our goal is to establish a cheaper source of pluripotent stem cell-derived hepatocytes, because a large number of hepatocytes are required for the development of cell therapy, drug screening and disease modeling. The present study has identified a small molecule, methoxamine hydrochloride, that can induce hepatocyte-like cells from both mESCs and hiPSCs by replacing the combination treatment of HGF and OsM, which has been used in most conventional hepatic differentiation protocols. In order to optimize the differentiation protocol using methoxamine, we examined various concentrations from 0.001 μM to 100 μM and found that the optimal concentration was 1 μM. We also found the diminished differentiation inducing potency of methoxamine at 100 μM due to its toxicity.

Tasmin *et al*. reported a hepatic differentiation protocol using a small molecule, SB431542 (1 μM), and 1% DMSO at Stage 3 without growth factors^13^. Siller *et al*. also developed a small molecule-based hepatic differentiation method consisting of three stages using only small molecules without growth factors^17^. They used a combination of two compounds, 100 nM Dihexa and 100 nM dexamethasone, at Stage 3. Compared with the inducing factors used in Stage 3 of these two methods, the factor in Stage 3 of our protocol, methoxamine hydrochloride (1 μM), is the cheapest. Our calculation indicates that the total cost of Stage 3 factors is 2.26 × 10^−2^ USD by Tasmin *et al*., 5.81 × 10^−4^ USD by Siller *et al*. and 1.52 × 10^−4^ USD by our method per well of a 24-well plates and 500 μl volume of culture medium. Assuming that a huge number of hepatocytes differentiated from hESCs/hiPSCs would be needed for clinical or pharmaceutical purposes, the cost savings at Stage 3 are significant. Moreover, the two reports used hiPSC and hESC lines, whereas our protocol was confirmed in human and mouse pluripotent stem cells, indicating greater robustness that could contribute to basic research as well as clinical application.

The limitations of our differentiation methods include the diminished differentiation efficiency of hepatocyte-like cells with long term cultures beyond day 20. Another limitation is that the hepatocyte-like cells obtained by our differentiation protocol are functionally immature, as evidenced by the lower expression levels of hepatocyte markers, such as multiple CYP enzymes, compared with primary human adult hepatocytes. Future studies should improve the differentiation protocol to more efficiently generate functionally mature hepatocytes. Nevertheless, our induction protocol for hepatocyte-like cells has broad utility and was shown applicable to both a mESC line and multiple hiPSC lines, suggesting its possible application to hiPSCs from liver disease patients.

In general, information about the target molecules of hit compounds allows for detailed study of the developmental or differentiation signalling mechanisms^15,18^. In the present study, the identification of methoxamine hydrochloride as a hepatic inducer led to the elucidation of novel mechanisms that signal through α1-adrenergic receptors for the differentiation of hepatoblasts into hepatocyte-like cells, and that the α1-adrenergic signalling pathways may work downstream of HGF and OsM and activate STAT3 pathways. This mechanistic discovery is in addition to lowering the cost and improving the stability of the differentiation method.

In summary, we have identified a small molecule, methoxamine hydrochloride, that can induce hepatocyte-like cells from both mESCs and hiPSCs through signalling downstream of HGF and OsM and by activating STAT3 pathways. These chemically-induced hepatocyte-like cells might be new cell sources for cell therapy, disease modelling and drug screening.

## Materials and Methods

### Study approval

All experimental protocols were carried out in accordance with the relevant guidelines and approved by the relevant committees. The use of hiPSCs was approved by the Ethics Committee of Kyoto University, and informed consent was obtained from all donor subjects from which hiPSC lines were generated in accordance with the Declaration of Helsinki.

### Cell culture

hiPSCs (201B6, 585A1, 604B1, 648A1, 648B1 and 610B1) and hESCs (KhES3) were grown on feeder layers of mitomycin C-treated SNL cells in media containing Primate ES medium (ReproCELL) supplemented with 500 U/ml penicillin/streptomycin (PS, Thermo Fisher Scientific) and 4 ng/ml recombinant human basic fibroblast growth factor (bFGF, Wako). For routine passaging, hiPSC colonies were dissociated by an enzymatic method with CTK dissociation solution consisting of 0.25% trypsin (Thermo Fisher Scientific), 0.1% collagenase IV (Thermo Fisher Scientific), 20% knockout serum replacement (KSR, Thermo Fisher Scientific) and 1 mM CaCl_2_ in PBS and split at a ratio between 1:3 and 1:6. mESCs (D3 cells) were maintained on feeder layers of mitomycin C-treated SNL cells in DMEM (Nacalai Tesque) supplemented with 15% fetal bovine serum (FBS, Thermo Fisher Scientific), 500 U/ml PS, 0.1 mM non-essential amino acid (Thermo Fisher Scientific), 2 mM glutamine (Thermo Fisher Scientific), 0.55 mM 2-mercaptoethanol (Thermo Fisher Scientific) and leukaemia inhibitory factor. As a source of LIF, we used a conditioned medium (1:10,000 dilution) from Plat-E cell cultures that had been transduced with a LIF-encoding vector. mESCs were passaged with enzymatic dissociation using 0.25% trypsin/EDTA (Thermo Fisher Scientific). hiPSCs and mESCs were routinely tested for mycoplasma contamination.

### Differentiation protocols

hiPSC colonies grown on SNL feeder cells were first treated with CTK dissociation solution, dissociated to single cells by gentle pipetting after the treatment with Accutase (Innovative Cell Technologies) for 20 min and seeded on Matrigel-coated plates (BD Biosciences) at a density of 1.0 × 10^5^ cells/cm^2^ with Stage 1 medium containing RPMI1640 (Nacalai Tesque) supplemented with 1 × B27 supplement (Thermo Fisher Scientific), 500 U/ml PS, 100 ng/ml recombinant human/mouse/rat activin A (R&D Systems) and 1 μM CHIR99021 (Wako). On day 1, 0.5 mM sodium butyrate (Sigma) was added in the medium, and the cells were cultured for an additional 5–6 days. For Stage 2, the cells were cultured in Stage 2 medium containing Knockout-DMEM (Thermo Fisher Scientific), 20% KSR, 1 mM L-glutamine, 1% nonessential amino acids, 0.1 mM 2-mercaptoethanol and 1% DMSO (Sigma) for 6–7 days. For Stage 3, the cells were cultured in hepatocyte culture medium (Lonza) containing 20 ng/ml recombinant human hepatocyte growth factor (HGF, Peprotech) and 20 ng/ml recombinant human Oncostatin M (OsM, Peprotech) for 7–8 days.

Hepatic differentiation of mESCs was performed according to the modified version of human protocol described above. The mESC colonies grown on SNL feeder cells were dissociated to single cells by gentle pipetting after treatment with 0.25% trypsin and seeded on gelatin-coated plates. After 10–20 minutes, culture supernatant containing mESCs were collected to remove feeder cells. The cells were seeded on Matrigel-coated plates at a density of 1.0–4.0 × 10^4^ cells/cm^2^ with Stage 1 medium containing RPMI1640 supplemented with 1 × B27 supplement, 500 U/ml PS, 100 ng/ml activin A and 1 μM CHIR99021. On day 1, 0.5 mM NaB was added in the medium, and the cells were cultured for an additional 5 days. For Stage 2, the cells were cultured in Knockout-DMEM containing 20% KSR, 1 mM L-glutamine, 1% nonessential amino acids, 0.1 mM 2-mercaptoethanol and 1% DMSO for 6 days. For Stage 3, the cells were cultured in hepatocyte culture medium containing 20 ng/ml HGF and 20 ng/ml OsM for 8 days.

### Chemical screening

hiPSCs (201B6) were first differentiated into definitive endoderm cells using 7 days of Stage 1 treatment described above. The cells were then dissociated with Accutase, seeded on 384-well plates at a density of 1.5 × 10^4^ cells/well in 100 μl of Stage 2 medium and incubated for 7 days to induce the differentiation into hepatoblasts. Next, the cells were treated with Stage 3 medium containing individual tested compounds or positive control (20 ng/ml HGF and 20 ng/ml OsM) using a robotic dispenser, Biomek 3000 (Beckman Coulter), and cultured for an additional 7 days by changing the medium with the compounds or growth factors after 4 days of treatment. Then, the cells were subjected to anti-ALBUMIN immunostaining, and the number and induction rate of ALBUMIN^+^ cells were examined using an image analyser, In Cell Analyzer 2000 (GE Healthcare).

### Flow cytometry and cell sorting

The cells were treated with 10 μM Y-27632 (Wako), incubated with Accumax (Innovative Cell Technologies) for 30 mins at 37 °C and dissociated by pipetting in PBS/2% FBS. Dead cells stained with Propidium-iodide (1 μg/ml, Wako) were excluded from the analysis. The cells were analyzed and sorted using a FACS Aria II cell sorter. The isolated cells were collected into PBS/2% FBS containing 10 μM Y-27632.

### BAC recombineering

The human BAC clone RP11-980A6, which contains all of the exons of the *ALBUMIN* gene and extends from 69.3 kb upstream to 92.8 kb downstream of the gene locus, was purchased from BACPAC Resource Center at Children’s Hospital, Oakland Research Institute. Recombineering was performed as previously described^29^. Briefly, the BAC clone was introduced into *E*. *coli* strain DH10B. To construct the targeting vector, we designed two primers that have short sequences of the homologous recombination regions of the *ALBUMIN* gene with the 5′ or 3′ end of EGFP-pA-PNL sequence and performed genomic PCR using KOD Plus Neo polymerase (TOYOBO) according to the manufacturer’s protocol. The primers used for genomic PCR were as follows: hALBUMIN-EGFP-S, TAATTTCCCTCCGTTTGTCCTAGCTTTTCTCTTCTGTCAACCCCACACGCCTTTGGCACAATGGTGAGCAAGGGCGAGGA; and hALBUMIN-PNL-AS, ATAGAAAAATGGATTTCTTACGTGCATCTCGACGAAACACACCCCTGGAATAAGCCGAGCGTCGACGGCGAGCTCAGACG. Then, the targeting vector, which was an EGFP-pA-PNL cassette containing 5′ and 3′ homology arms, was electroporated into DH10B containing BAC RP11-980A6 and activated recombinases. The transformed bacteria were plated on LB plates with appropriate antibiotics and incubated overnight at 37 °C. Selected clones were picked and subjected to PCR to confirm whether the EGFP-pA-PNL cassette was integrated into the *ALBUMIN* endogenous locus through successful homologous recombination.

### Genetic modification of hiPSCs

Electroporation was performed as previously described^35^. The human *ALBUMIN-EGFP-pA-PNL* BAC vector was linearized by restriction enzymes and sterilized by ethanol precipitation. hiPSCs (201B6) were treated with 10 μM Y-27632 overnight and trypsinized. Cells were then centrifuged and re-suspended in PBS. A total of 30 μg of linearized DNAs was added into the resuspended hiPSCs. The cells were subjected to a single 250 V, 500 mF pulse (Gene pulser CE, Bio-Rad) at room temperature and plated on feeder layers of mitomycin C-treated SNL cells. Antibiotic selection was applied two days after electroporation.

### Removal of PGK-Neo cassette

The hiPSCs with the *ALBUMIN* transgene were treated with 10 μM Y-27632 overnight and trypsinized. Cells re-suspended in PBS were electroporated with 30 μg of pCXW-Cre-Puro as described above, and plated on feeder layers of mitomycin C-treated SNL cells. Antibiotic selection was applied a week after electroporation.

### RNA sequencing

Total RNA samples of hiPSCs and hiPSC-derived differentiated cells were extracted with RNeasy kit (Qiagen). Total RNA samples of adult and fetal livers were obtained from Clontech Laboratories Inc. One hundred ng of total RNA was subjected to library preparation using TruSeq Stranded Total RNA with Ribo-Zero Gold LT Sample Prep Kit (Illumina), according to the manufacturer’s instruction. The libraries were sequenced in 100 cycle Single-Read mode of HiSeq2500. All sequence reads were extracted in FASTQ format using BCL2FASTQ Conversion Software 1.8.4 in the CASAVA 1.8.2 pipeline. The sequence reads were mapped to hg19 reference genes downloaded on 25th April 2014 using Tophat v2.0.14. Calculation of the gene expression values and normalization were performed by RPKMforgenes (10th Dec. 2012), and the expression levels were represented by log_2_(RPKM+1). A heatmap of gene expressions was generated by heatmap.2 function of the gplots library in R 3.2.1. Principle component analysis (PCA) was performed by prcomp function in R 3.2.1.

### Western blot analysis

Total cellular proteins were extracted using cell lysis buffer (Wako). The protein concentrations of the lysates were determined by a Bradford assay. Samples containing 50 μg of protein were separated by 4–20% sodium dodecyl sulfate-polyacrylamide gel electrophoresis (Bio-Rad) and transferred to a polyvinylidene difluoride membrane (Bio-Rad). After blocking with 5% skim milk in PBS/0.1% Tween20, the membrane was incubated with primary antibodies against Phospho-Stat3 (Cell Signaling), Stat3 (Cell Signaling), and β-Actin (Sigma-Aldrich) (Table S3) overnight at 4 °C, followed by incubation with horseradish peroxidase-conjugated anti-mouse/rabbit secondary antibodies (GE Healthcare) at room temperature for 1 hour. ECL Prime Blotting Detection Reagent (GE Healthcare) was used for chemiluminescent detection. The band intensity was quantified using image analysis software Multi Gauge (Fujifilm).

### Statistical analysis

Statistical analyses were performed using GraphPad PRISM software version 7 (GraphPad Software Inc). The number of replicates, statistical tests and the test results are shown in the figure legends. All statistical tests were two-tailed. Sample size was not pre-defined by power calculations. No experimental samples were excluded from the statistical analyses. No randomization or investigator blinding were performed in the experiments and analyses of this study. When representative data are shown, the results were reproduced using at least three independent experiments. P values < 0.05 were considered to be statistically significant.

### Data availability

The NCBI GEO accession number for the RNA sequencing data reported in this paper is GSE83480.

## Electronic supplementary material

Supplementary Information
