# Supplementary material for: Adrenergic receptor agonists induce the differentiation of pluripotent stem cell-derived hepatoblasts into hepatocyte-like cells
Source: Sci Rep. 2017 Dec 1;7:16734. doi: 10.1038/s41598-017-16858-5 (PMC5711806; doi:10.1038/s41598-017-16858-5)
Supplement: Supplementary file 1 — Supplementary Information [file 41598_2017_16858_MOESM1_ESM.doc]

**Supplemental Information**

**Adrenergic receptor agonists induce the differentiation of pluripotent stem cell-derived hepatoblasts into hepatocyte-like cells**

**Maki Kotaka, Taro Toyoda, Katsutaro Yasuda, Yuko Kitano, Chihiro Okada, Akira Ohta, Akira Watanabe, Motonari Uesugi and Kenji Osafune**

**Supplemental Figure S1**

**
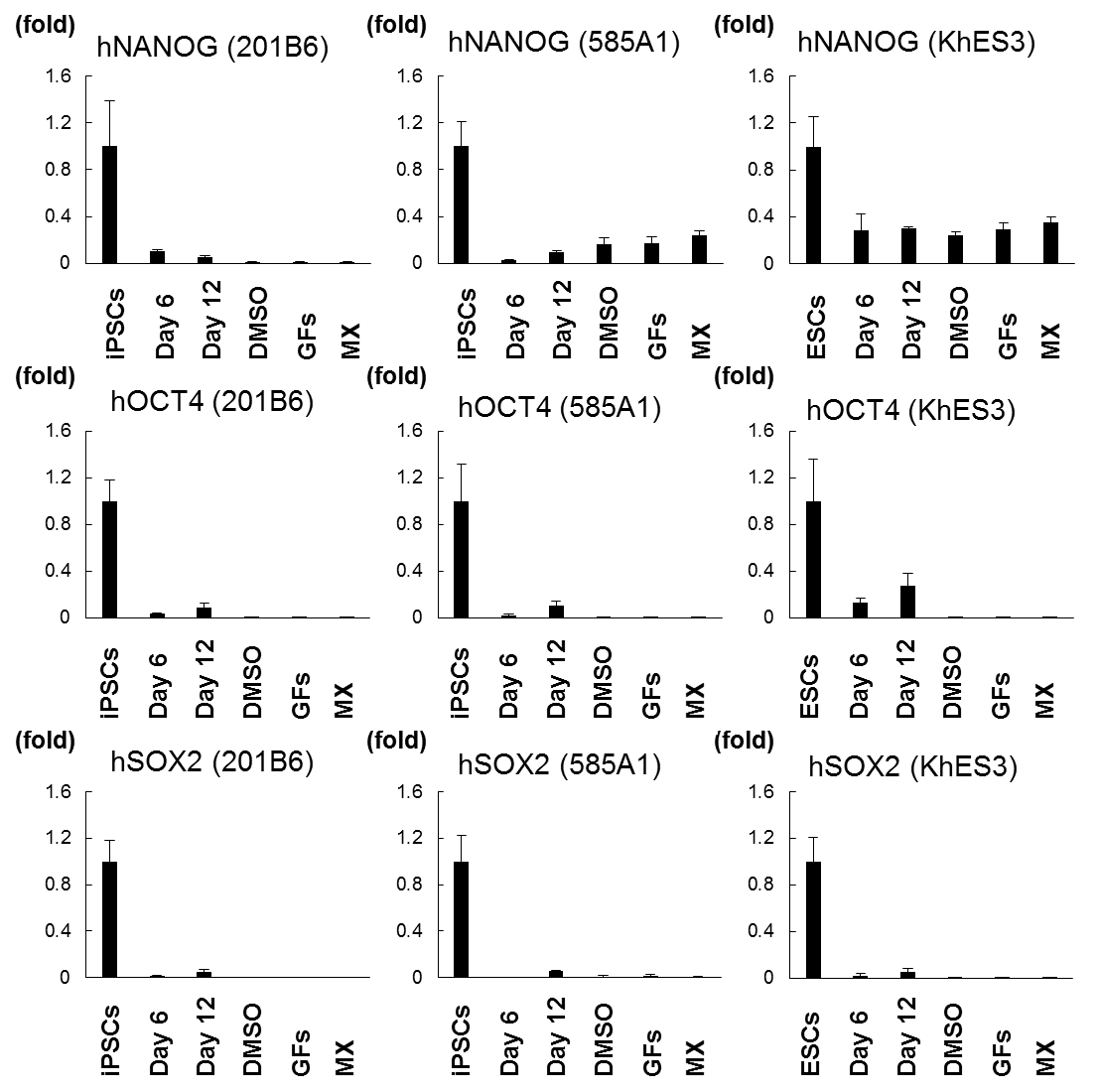
**

**Figure S1. Temporal expression patterns of pluripotency markers.**

qRT-PCR analyses of the expression of pluripotency marker genes in undifferentiated hiPSCs and hiPSC-derived differentiated cells on days 6, 12 (Stage 2, day 6) and 20 (Stage 3, day 8) after treatment with DMSO, a combination of HGF and OsM (GFs) or methoxamine alone (MX). Values were normalized to those of undifferentiated hiPSCs.

**Supplemental Figure S2**

**
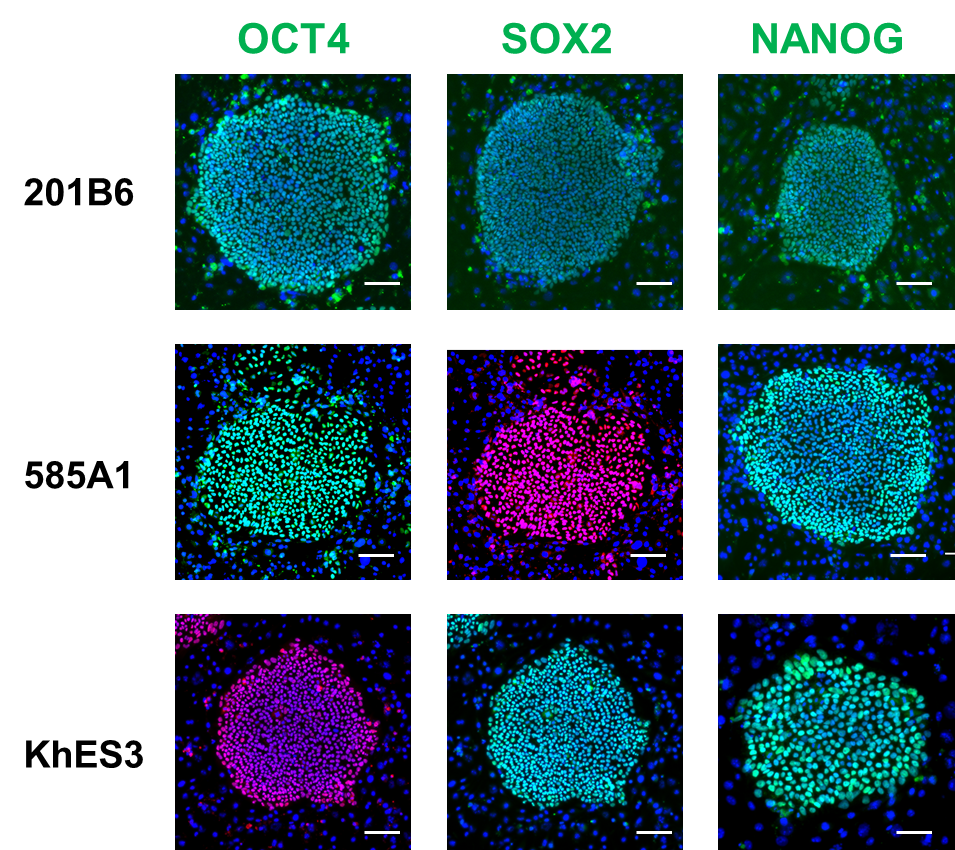
**

**Figure S2. Protein expression of pluripotency marker genes.**

Immunostaining analyses of pluripotency markers, OCT4, SOX2 and NANOG, in two hiPSC lines (201B6 and 585A1) and one hESC line (KhES3). Scale bars, 100 μm.

**Supplemental Figure S3**

**
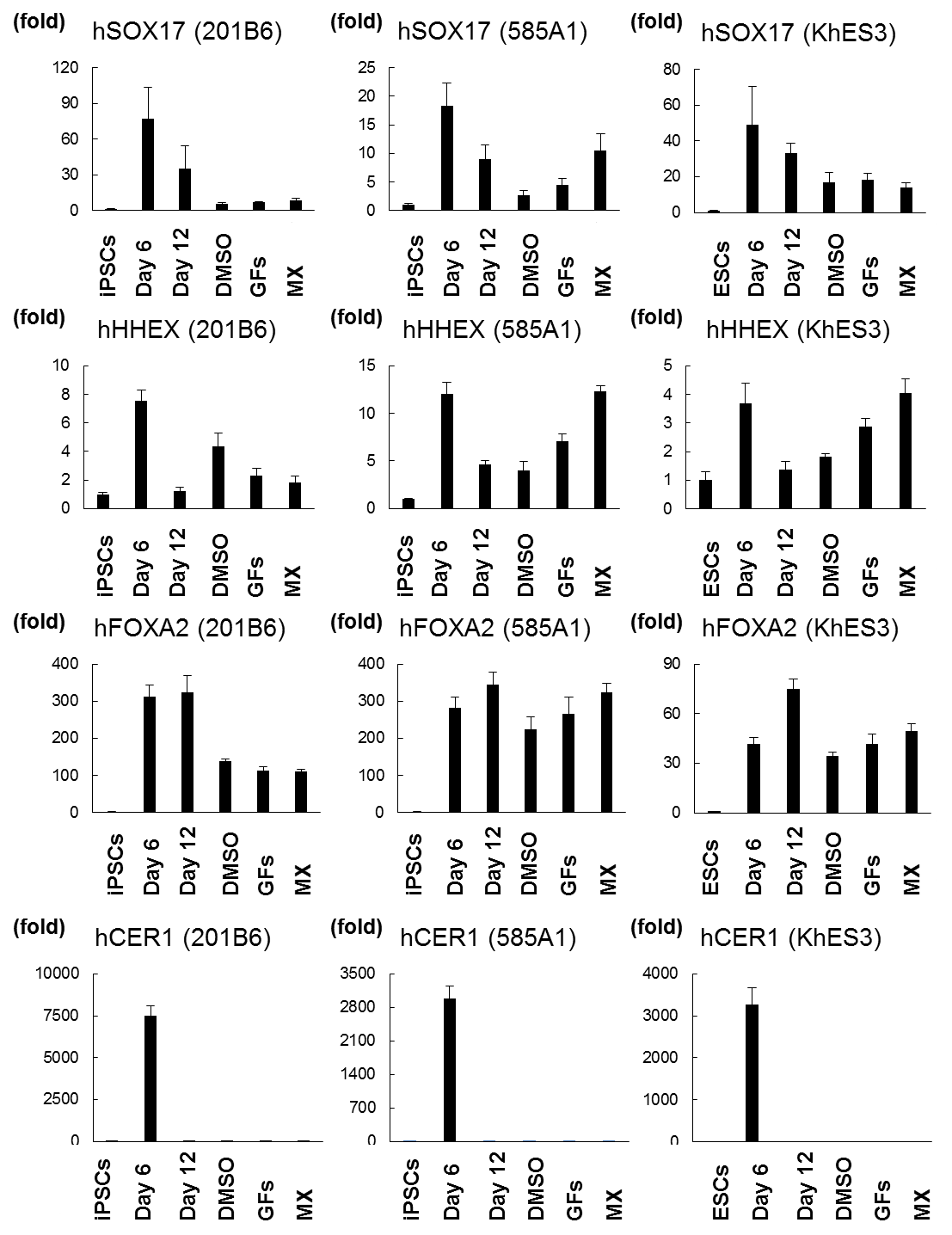
**

**Figure S3. Temporal expression patterns of definitive endoderm markers.**

qRT-PCR analyses of the expression of definitive endoderm markers in undifferentiated hiPSCs and hiPSC-derived differentiated cells on days 6, 12 (Stage 2, day 6) and 20 (Stage 3, day 8) after treatment with DMSO, a combination of HGF and OsM (GFs) or methoxamine alone (MX). Values were normalized to those of undifferentiated hiPSCs. CER1: Cerberus 1.

**Supplemental Figure S4**

**
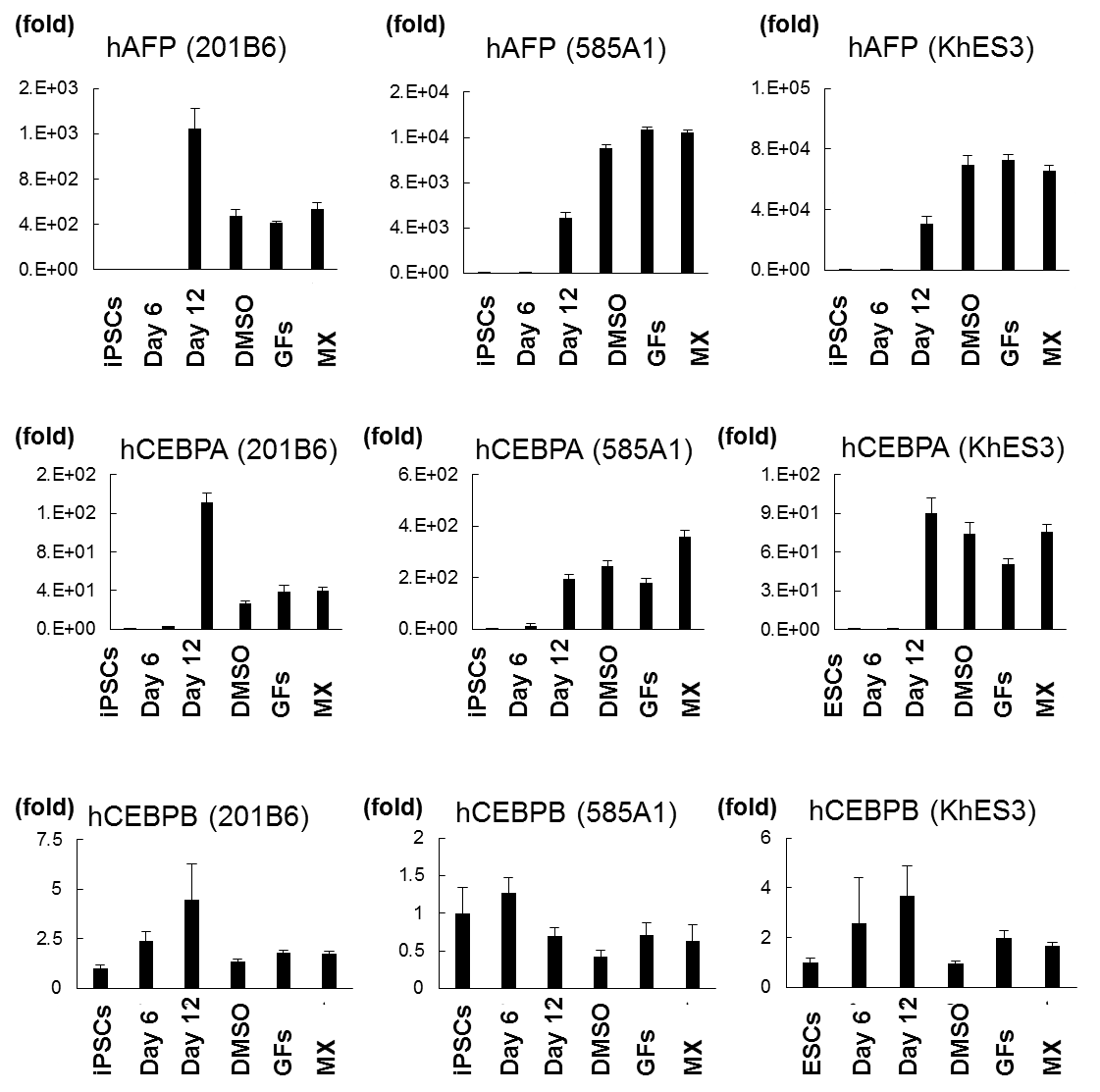
**

**
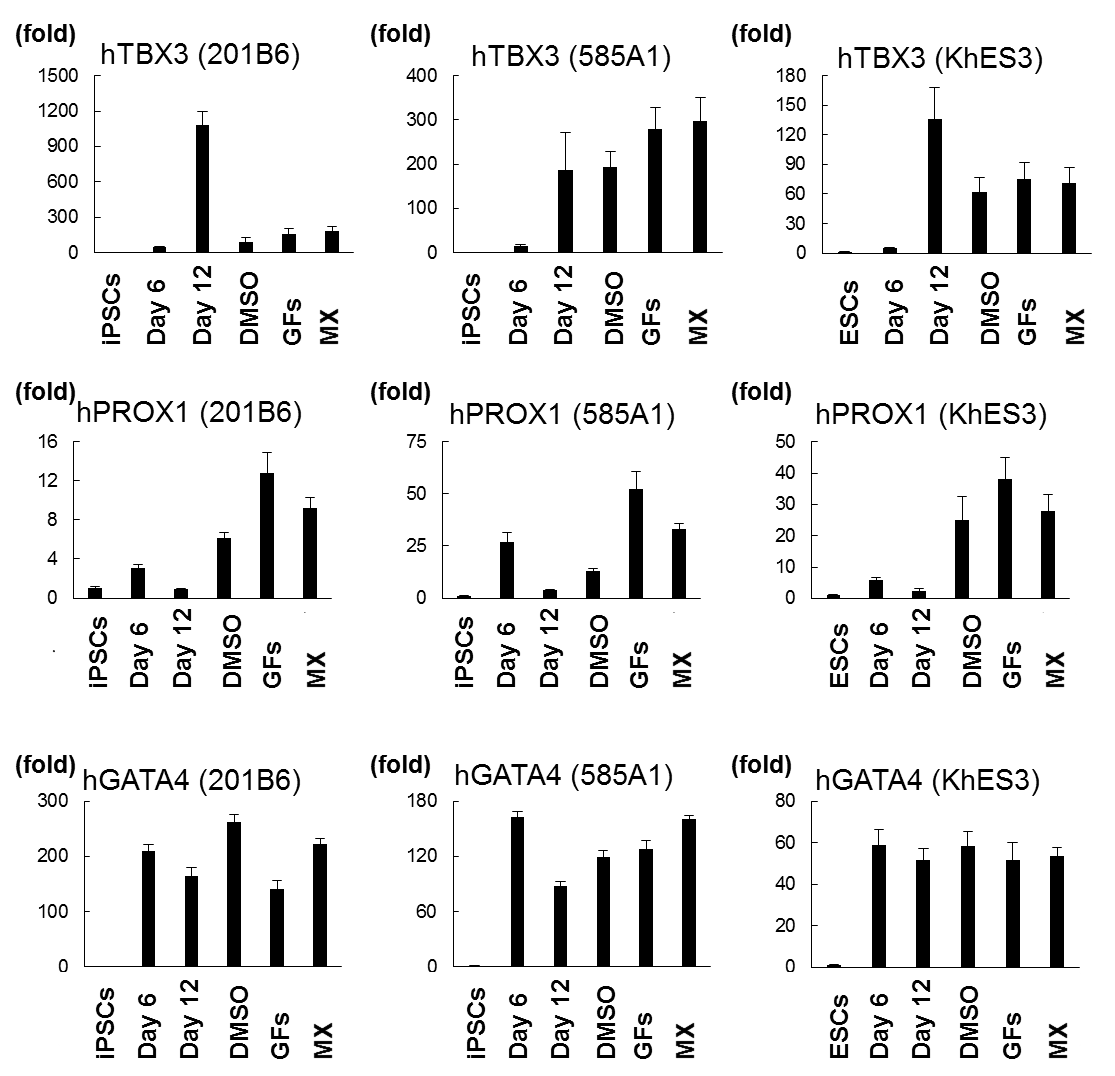
**

**Figure S4. Temporal expression patterns of hepatoblast markers.**

qRT-PCR analyses of the expression of hepatoblast markers in undifferentiated hiPSCs and hiPSC-derived differentiated cells on days 6, 12 (Stage 2, day 6) and 20 (Stage 3, day 8) after treatment with DMSO, a combination of HGF and OsM (GFs) or methoxamine alone (MX). Values were normalized to those of undifferentiated hiPSCs. AFP: alpha-fetoprotein, CEBP: CCAAT/enhancer binding protein.

**Supplemental Figure S5**

**
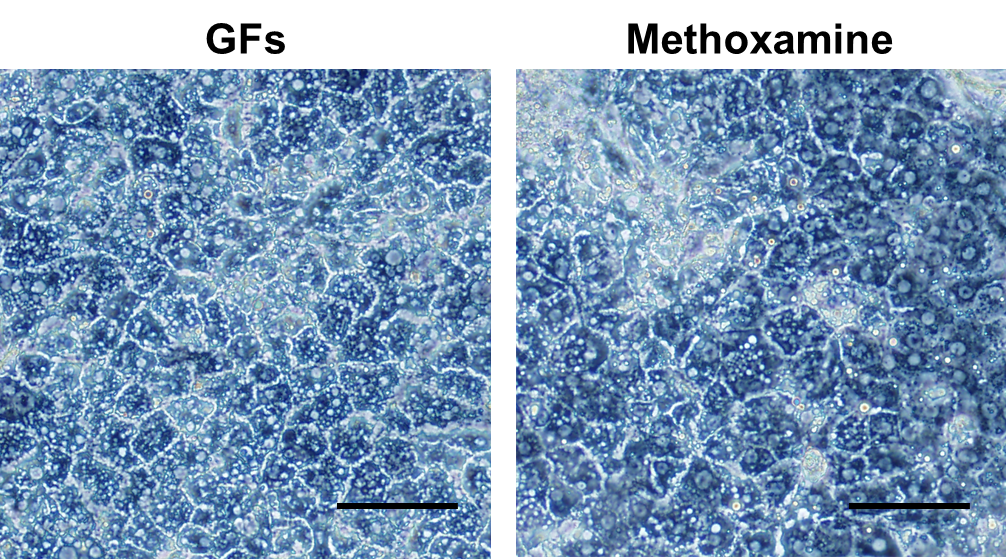
**

**Figure S5. Morphology of hepatocyte-like cells induced from hiPSCs.**

Phase contrast images of hiPSC-derived hepatocyte-like cells on culture day 20 induced with a combination of HGF and OsM (GFs, left) or methoxamine alone (right). Scale bars, 100 μm.

**Supplemental Figure S6**

**
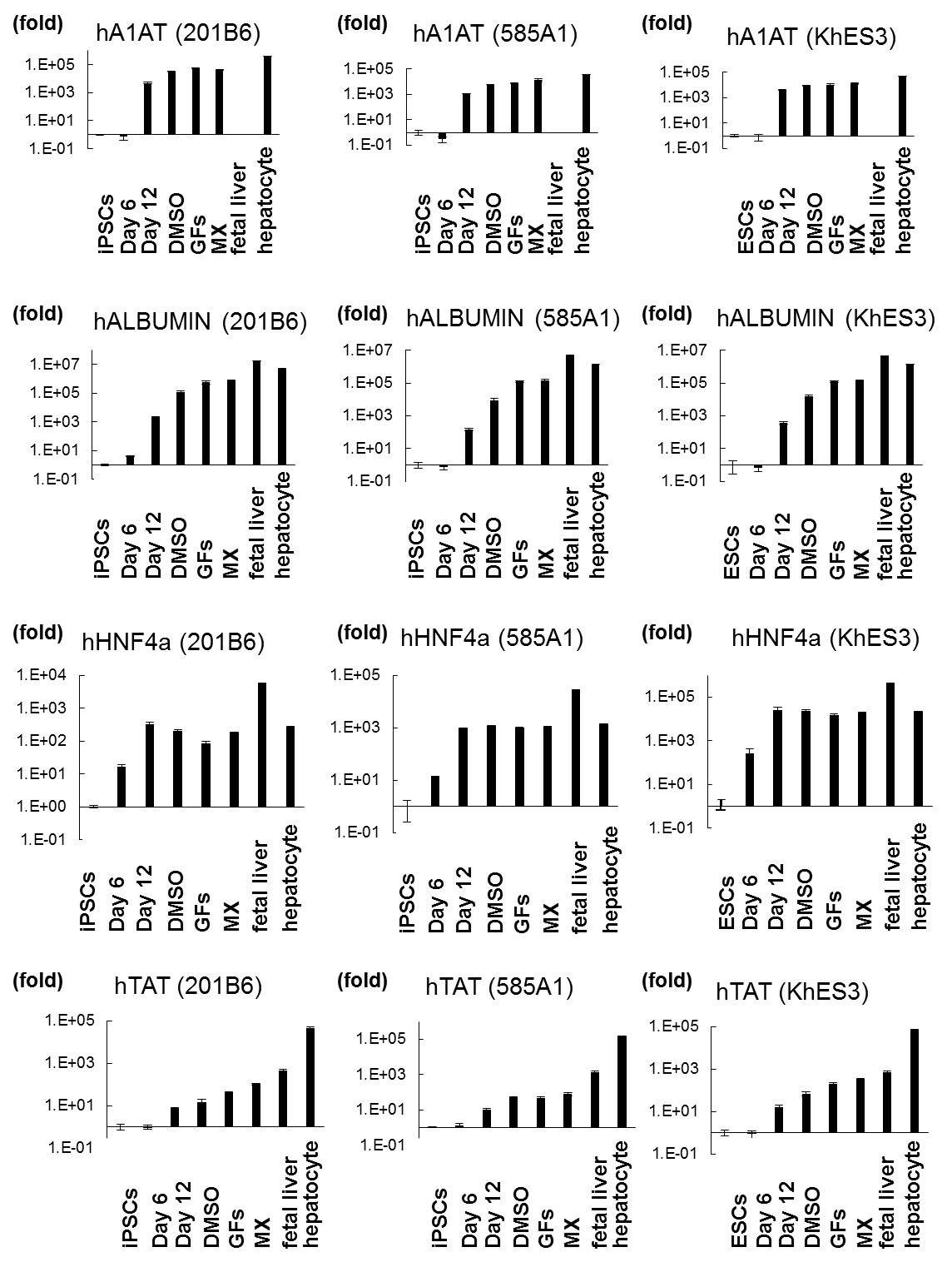
**

**
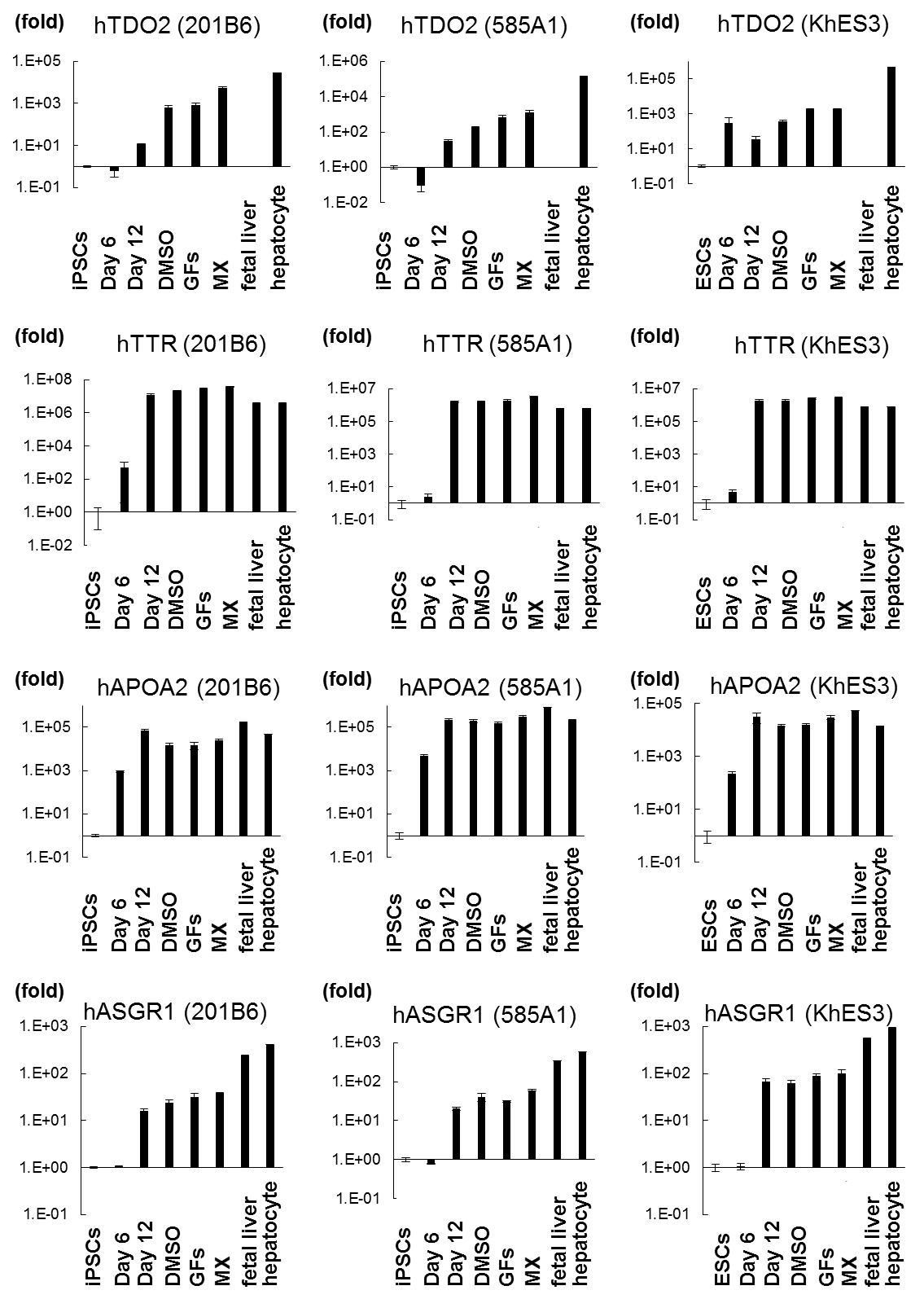
**

**
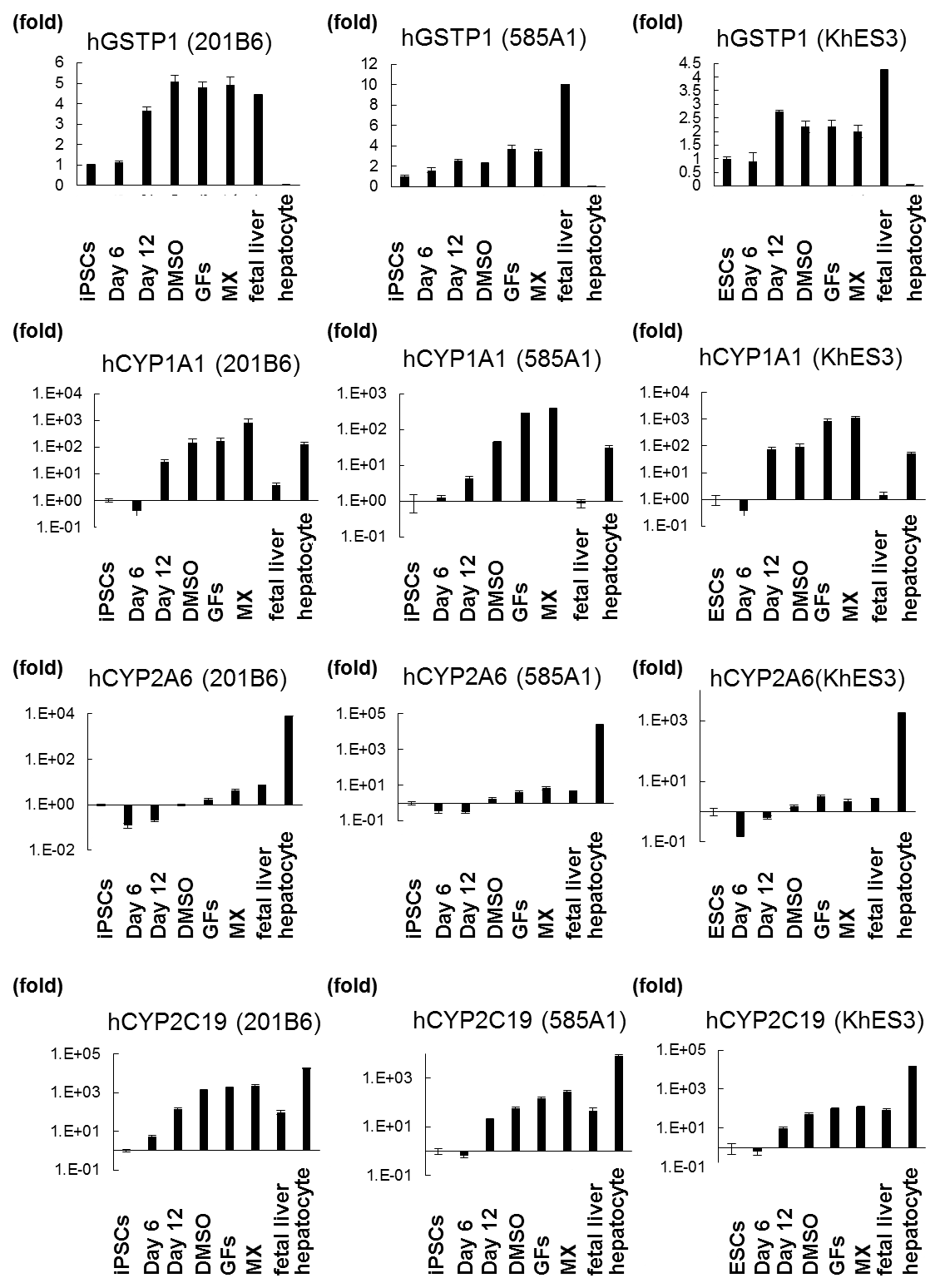
**

**
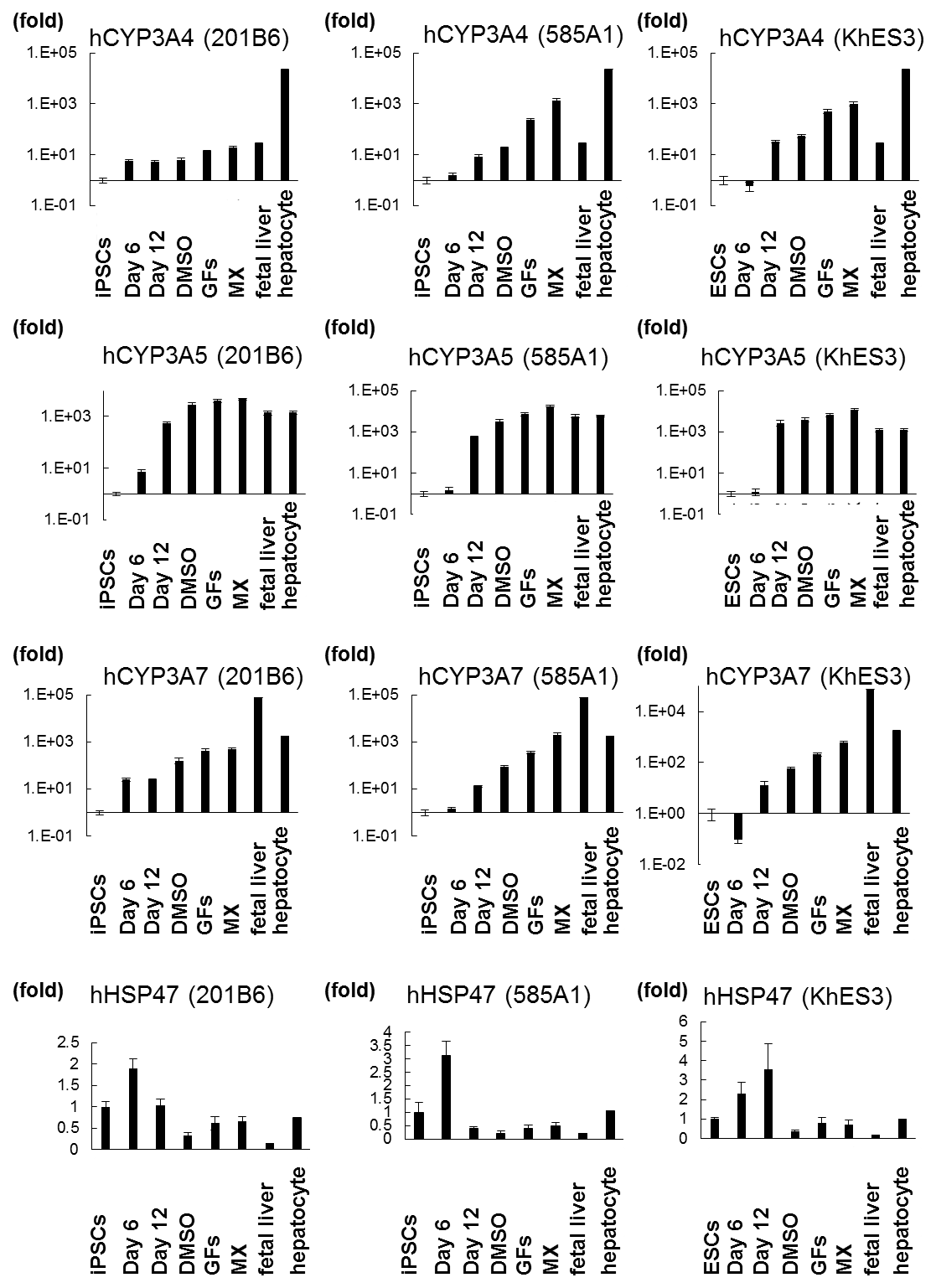
**

**Figure S6. Temporal expression patterns of hepatocyte markers.**

qRT-PCR analyses of the expression of hepatocyte markers in undifferentiated hiPSCs and hiPSC-derived differentiated cells on days 6, 12 (Stage 2, day 6) and 20 (Stage 3, day 8) after treatment with DMSO, a combination of HGF and OsM (GFs) or methoxamine alone (MX). Values were normalized to those of undifferentiated hiPSCs. A1AT: α1-antitrypsin, TAT: tyrosine aminotransferase, TDO2: tryptophan2,3-dioxygenase 2, TTR: transthyretin**,** APOA2: apolipoprotein A 2, ASGR1: asialoglycoprotein receptor 1, GSTP1: glutathione S-transferase protein 1, CYP: cytochrome P450, HSP: heat-shock protein.

**Supplemental Figure S7**

**Full unedited gel for Figure 3B**

**
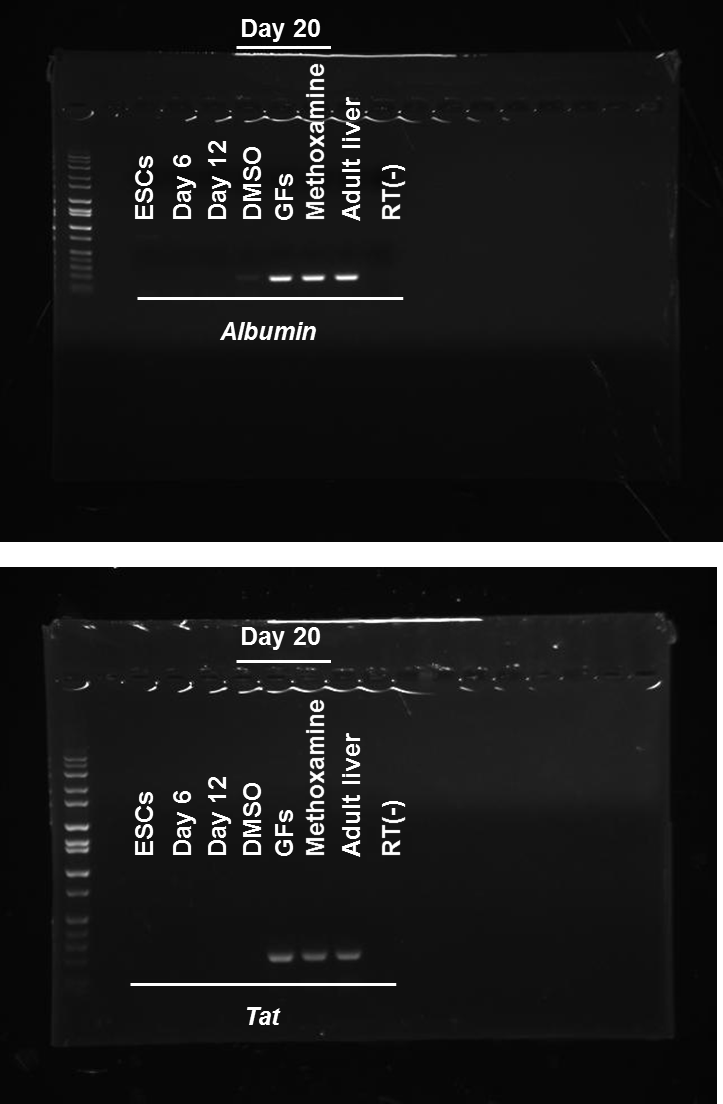
**

**
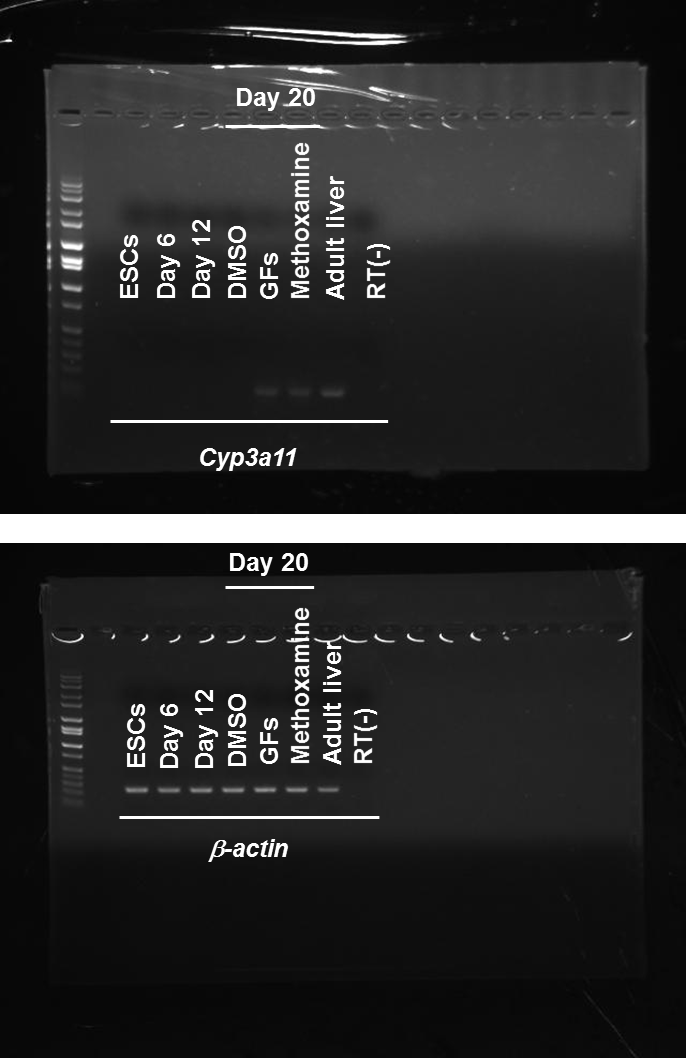
**

**Supplemental Figure S8**

**Full unedited gel for Figure 4D**

**
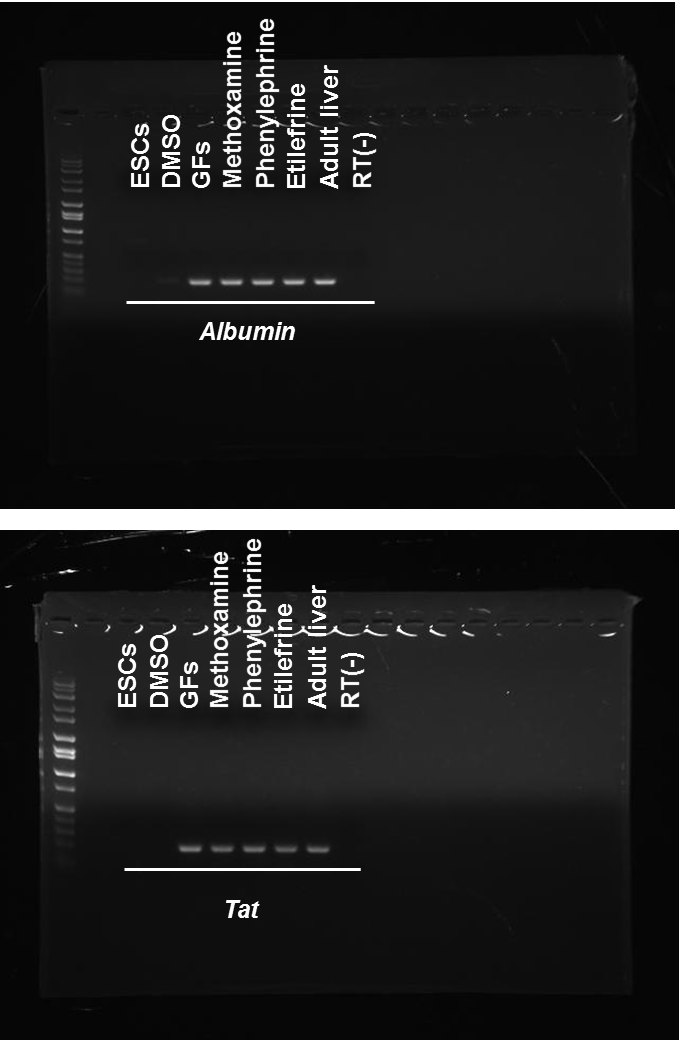
**

**
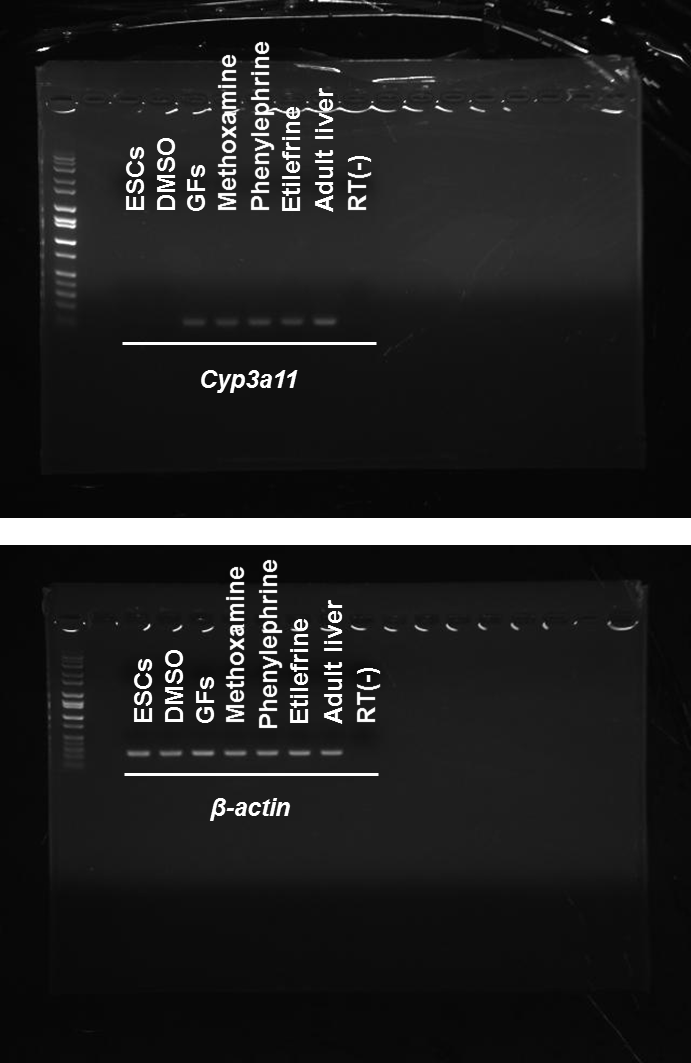
**

**Supplemental Figure S9**

**Figure S9. Whole genome sequencing identified the genomic breakpoint of the *ALB*-coding region on BAC vector with chr9.** (A) The number of sequenced reads on the *ALB* genomic region (chr4:74,025,232-74,426,389). A genomic region with increased copy number indicates the inserted coding region in BAC vector including *ALB* gene (red arrow). (B) Breakpoints at chr4 (left and middle) and chr9 (right). All sequence reads were mapped to a putative reference sequence of the chimera genome, of which the junctions were chr4:74,328,970 and chr9:31,965,964 (left) and chr9:31,965,960 and chr4:74,207,043 (right). Reads in gray have paired sequences on the same chromosome; reads in blue have the paired sequences on the other chromosome. (C) Mapping of sequenced reads to the reference chimera sequence. Two gray bars connected by the black dashed line indicate paired sequenced reads. Chimera reads indicate a deletion of GCC nucleotides from 31,965,961 to 31,965,963 in chromosome 9 and insertion of AAA instead of deletion.

**Supplemental Figure S10**

**
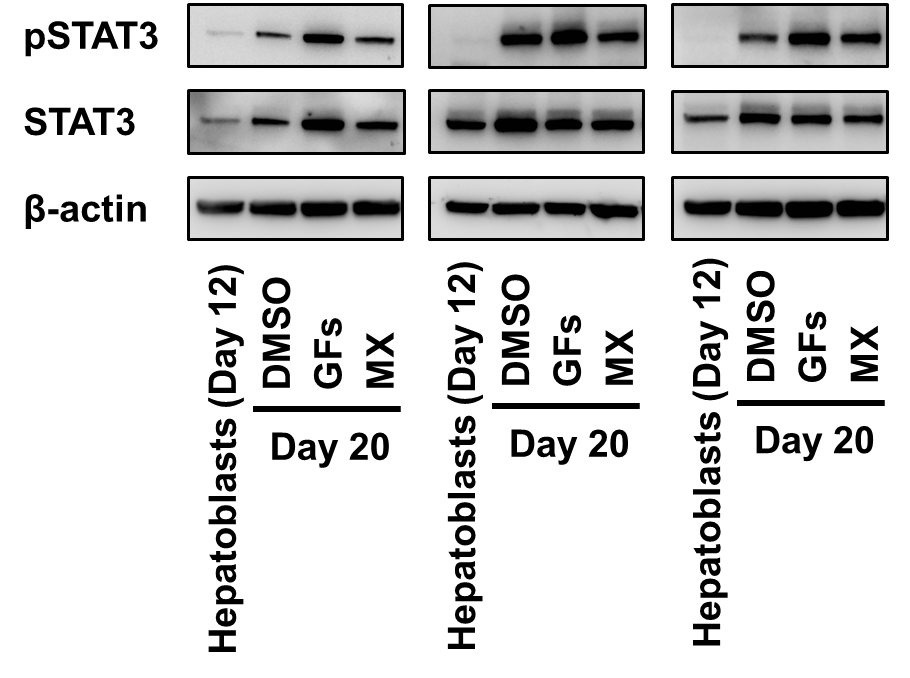
**

**Figure S10. Phosphorylation levels of STAT3 in hiPSC-derived hepatocyte-like cells.**

Western blot analyses of the protein expression of STAT3 and phosphorylated STAT3 in hiPSC-derived hepatocyte-like cells on culture day 20 after treatment with DMSO, a combination of HGF and OsM (GFs) or methoxamine alone (MX). The data from three independent experiments are shown.

**SUPPLEMENTAL TABLES**

**Table S1. Pathway analysis. Related to Figure 6.**

|  | Pathway | | Symbol | | Fold Change Methoxamine vs. HGF+OsM | |
| --- | --- | --- | --- | --- | --- | --- |
|  | 1. Statin Pathway | |  | |  | |
|  | cytochrome P450, family 7, subfamily A, polypeptide 1 | | CYP7A1 | | * | |
|  | apolipoprotein C-III | | APOC3 | | 1.200 | |
|  | apolipoprotein C-II | | APOC2 | | 1.289 | |
|  | apolipoprotein A-IV | | APOA4 | | 1.228 | |
|  | lipase, hepatic | | LIPC | | 2.283 | |
|  | apolipoprotein C-I | | APOC1 | | 1.147 | |
|  | 2. ACE-Inhibitor Pathway | |  | |  | |
|  | kininogen 1 | | KNG1 | | 1.212 | |
|  | angiotensinogen (serpin peptidase inhibitor, clade A, member 8) | | AGT | | 0.959 | |
|  | 3. Blood Clotting Cascade | |  | |  | |
|  | coagulation factor XIII, B polypeptide | | F13B | | * | |
|  | fibrinogen alpha chain | | FGA | | 0.604 | |
|  | coagulation factor II (thrombin) | | F2 | | 1.055 | |
|  | plasminogen | | PLG | | 1.090 | |
|  | coagulation factor V (proaccelerin, labile factor) | | F5 | | 1.079 | |
|  | lipoprotein, La(a) | | LPA | | 0.961 | |
|  | coagulation factor XI | | F11 | | 0.116 | |
|  | 4. Glycolysis and Gluconeogenesis | |  | |  | |
|  | aldolase B, fructose-bisphosphate | | ALDOB | | 1.523 | |
|  | glucose-6-phosphatase, catalytic subunit | | G6PC | | 0.643 | |
|  | 5. Irinotecan Pathway | |  | |  | |
|  | cytochrome P450, family 3, subfamily A, polypeptide (CYP3A4) | | CYP3A4 | | * | |
|  | cytochrome P450, family 3, subfamily A, polypeptide (CYP3A5) | | CYP3A5 | | 1.082 | |
|  | 6. Steroid Biosynthesis | |  | |  | |
| cytochrome P450, family 17, subfamily A, polypeptide 1 | | CYP17A1 | | * | |  |
| cytochrome P450, family 11, subfamily A, polypeptide 1 | | CYP11A1 | | 0.430 | |  |
| hydroxy-delta-5-steroid dehydrogenase, 3 beta- and steroid delta-isomerase 2 | | HSD3B2 | | 0 | |  |
| cytochrome P450, family 21, subfamily A, polypeptide 2 | | CYP21A2 | | 0.629 | |  |
| cytochrome P450, family 11, subfamily B, polypeptide 1 | | CYP11B1 | | * | |  |
| cytochrome P450, family 11, subfamily B, polypeptide 2 | | CYP11B2 | | * | |  |
| cytochrome P450, family 19, subfamily A, polypeptide 1 | | CYP19A1 | | 2.746 | |  |
| hydroxysteroid (17-beta) dehydrogenase 3 | | HSD17B3 | | 0.562 | |  |
| 7. Adrenaline Biosynthesis | |  | |  | |  |
| tyrosine hydroxylase | | PH | | * | |  |
| dopa decarboxylase (aromatic L-amino acid decarboxylase) | | DDC | | 1.004 | |  |
| dopamine beta-hydroxylase | | DBH | | 1.825 | |  |
| phenylethanolamine N-methyltransferase | | PNMT | | * | |  |

*, Denominator is zero.

**Table S2.** **Primer sequences used in this study.**

| Gene name | Primer (forward / reverse ; 5’ to 3’) |
| --- | --- |
| *hGAPDH* | GAGTCAACGGATTTGGTCGT/GACAAGCTTCCCGTTCTCAG |
| *hOCT4* | GGAAGCCCAAGAACCTGAAT/GTTGCTGGAGTTGCTGGAA |
| *hSOX2* | ACCAGCGCATGGACAGTTAC/CCGTTCATGTAGGTCTGCGA |
| *hNANOG* | CAATGGTGTGACGCAGGGAT/TGCACCAGGTCTGAGTGTTC |
| *hSOX17* | CAGCAGAATCCAGACCTGCA/GTCAGCGCCTTCCACGACT |
| *hHHEX* | ACCATCGAGCTGGAGAAGAA/TGCTTTGAGGGTTCTCCTGT |
| *hCER1* | GGGGTCATCTTGCCCATCAA/CCCGCATTTCCCAAAGCAAA |
| *hFOXA2* | TGTGTATTCTGGCTGCAAGG/CCTGCAACCAGACAGGGTAT |
| *hCEBPA* | GCAAACTCACCGCTCCAATG/CTTCTCTCATGGGGGTCTGC |
| *hCEBPB* | TTTGTCCAAACCAACCGCAC/GCATCAACTTCGAAACCGGC |
| *hAFP* | AAATGCGTTTCTCGTTGCTT/GCCACAGGCCAATAGTTTGT |
| *hHNF4A* | TGCGACTCTCCAAAACCCTC/ATTGCCCATCGTCAACACCT |
| *hPROX1* | TTCCACTGACCAGACAGAAG/TGGGCTCTGAAATGGATAGG |
| *hTBX3* | AGTCGGGAAGGCGAATGTTT/GGACATCCACTGTTCCCCAG |
| *hAAT* | TCGCTACAGCCTTTGCAATG/TTGAGGGTACGGAGGAGTTCC |
| *hALBUMIN* | CGCTATTAGTTCGTTACACCA/TTTACAACATTTGCTGCCCA |
| *hAPOA2* | GTCAAGAGCCCAGAGCTTCA/GCTGTGTTCCAAGTTCCACG |
| *hASGR1* | CACGTGAAGCAGTTCGTGTC/CGGAGCGAGAGAACCAGTAG |
| *hCYP1A1* | CCACCAAGAACTGCTTAGCC/CAGCTCCAAAGAGGTCCAAG |
| *hCYP1A2* | TCAAGCAATCCTCCCACTTC/ACATGGTGAAACCCCATCTC |
| *hCYP2A6* | GCTAGGAATCTTCCAGTTCACG/TCTTGGCTATGAAGTCCTCCAG |
| *hCYP3A4* | AAGACCCCTTTGTGGAAAAC/CGAGGCGACTTTCTTTCATC |
| *hCYP3A5* | CTCTCTGTTTCCAAAAGATACC/TGAAGATTATTGACTGGGCTG |
| *hCYP3A7* | AGATTTAATCCATTAGATCCAT/AGGCGACCTTCTTTTATCTG |
| *hCYP2B6* | GTGCGGAATTGTTCCTCTTC/AAGCGGATCTGGTATGTTGG |
| *hCYP2C19* | GAACACCAAGAATCGATGGACA/TCAGCAGGAGAAGGAGAGCATA |
| *hTAT* | ATCTCTGTTATGGGGCGTTG/TGATGACCACTCGGATGAAA |
| *hTDO2* | GACGGCTGTCATACAGAGCA/ACTCACAGTTGATCGCAGGT |
| *hTTR* | TGGAAGGCACTTGGCATCTC/TCCTTGGGATTGGTGACGAC |
| *hGSTP1* | CAGGGAGGCAAGACCTTCAT/GAGGTTCACGTACTCAGGGG |
| *hHSP47* | GGACTGCCATATATAGATCCCG/GCCTGCCTTTTTCATTCTGG |
| *hADRA1A* | AGCTTTTTGCAGGTCTGCTG/ATTCCCCTTTCCTCTGCATC |
| *hADRB2* | AAGGCAGCTCCAGAAGATTG/ATTCCCCTTTCCTCTGCATC |
| *GFP* | TGAACTTCAAGATCCGCCACA/TTCTCGTTGGGGTCTTTGCT |
| *mβ-actin* | TGCGTGACATCAAAGAGAAGC/GGATGTCAACGTCACACTTC |
| *mTat* | GGAGGAGGTCGCTTCCTATT/GCCACTCGTCAGAATGACATC |
| *mCyp3a11* | GGGACTCGTAAACATGAACTTTTT/CCATGTCGAATTTCCATAAACC |
| *mAlbumin* | GGTGTGTTTCGCCGAGAAGCAC/GGCGGCAGACTCATCGGC |
| *mAdra1a* | TCGACCGATACATTGGTGTG/TGGAGATGACCAAGGAAAGC |
| *mAdrb2* | AGCAATAGCAACGGCAGAAC/GTTCACAAAGCCTTCCATGC |

**Table S3. Primary antibodies used in this study.**

| Antigen | Dilution rate | Manufacturer |
| --- | --- | --- |
| ALBUMIN | 1 : 250 | Bethyl Laboratories, A80-229A |
| CYP3A4 | 1 : 100 | Proteintech, 18227-1-AP |
| CK18 | 1 : 250 | Invitrogen, 18-0158Z |
| AAT | 1 : 50 | Invitrogen, PA1-22860 |
| CYP1A2 | 1 : 100 | Proteintech 19936-1-AP |
| CYP2D6 | 1 : 100 | SIGMA-ALDRICH, AV41675 |
| Albumin | 1 : 250 | Bethyl Laboratories, A90-135 |
| Cyp1a2 | 1 : 100 | Proteintech 19936-1-AP |
| Cyp3a11 | 1 : 100 | Proteintech, 18227-1-AP |
| E-Cadherin | 1 : 250 | BD Biosciences, 610181 |
| NANOG | 1 : 500 | Cell Signaling, #4903 |
| SOX2 | 1 : 500 | Cell Signaling, #3579 |
| OCT3/4 | 1 : 500 | R&D, AF1759 |
| Phospho-Stat3 | 1 : 1000 | Cell Signaling, #9131 |
| Stat3 | 1 : 1000 | Cell Signaling, #9139 |
| β-Actin | 1 : 5000 | SIGMA-ALDRICH, A1978 |

**Table S4. Secondary antibodies used in this study.**

| Antigen | Fluorescent dyes | Manufacturer |
| --- | --- | --- |
| Goat IgG | Alexa fluor 488 | Life technologies A11055 |
| Rabbit IgG | Alexa fluor 488 | Life technologies A21206 |
| Mouse IgG | Alexa fluor 488 | Life technologies A21202 |
| Goat IgG | Alexa fluor 546 | Life technologies A11056 |

**Table S5. Inhibitors used in this study.**

| Adrenergic receptor | Inhibitor | Manufacturer |
| --- | --- | --- |
| α1 | prazosin | SIGMA P7791-50MG |
| α2 | yohimbine | SIGMA Y3125-1G |
| β1 | metoprolol | SIGMA M5391-1G |
| β2 | butoxamine | SIGMA B1385-50MG |

**Supplementary Materials and Methods**

**RT-PCR and real-time quantitative RT-PCR (qRT-PCR)**

Total RNA was isolated from triplicate samples in three independent experiments by a standard RNA isolation method using Trizol (ambion) followed by cDNA synthesis using standard protocols^1^. Briefly, first-strand cDNA was synthesized from 2 μg of total RNA using ReverTra Ace (TOYOBO). The cDNA samples were subjected to PCR amplification using a thermal cycler (Veriti 96 well Thermal Cycler, Applied Biosystems). PCR was performed using the Ex-Taq PCR kit (Takara) according to the manufacturer’s instructions. The PCR cycles were as follows: for *GAPDH* and *β-actin*, initial denaturation at 94 °C for 2.5 min, followed by 25 cycles of 94 °C for 30 s, 60 °C for 30 s, 72 °C for 30 s, and a final extension at 72 °C for 10 min. For the other genes, the cycles consisted of initial denaturation at 94 °C for 2.5 min, followed by 30-45 cycles of 94 °C for 30 s, 58-64 °C for 30 s, 72 °C for 30 s, and a final extension at 72 °C for 7 min. qPCR was performed using the Step One Plus Real-Time PCR System (Applied Biosystems) and SYBR Green PCR Master Mix (Takara). Denaturation was performed at 95 °C for 30 s followed by 45 cycles at 95 °C for 5 s and at 60 °C for 30 s. As recommended by the manufacturer, the threshold cycle method was used to analyze the data for gene expression levels, which were calibrated to that of the housekeeping gene *GAPDH*. The PCR reactions were performed in triplicate for each sample. The primer sets used in this study are shown in Table S2.

**Immunostaining**

The cells were fixed with 4% paraformaldehyde (PFA)/PBS for 20 min at 4 °C. After washing with PBS, the cells were blocked with 1% normal goat or donkey serum (Chemicon)/3% BSA (Nacalai Tesque)/PBST (PBS/0.25% Triton X-100) for 1 hour at room temperature. Primary antibodies were diluted in each blocking solution and incubated with samples overnight at 4 °C. Secondary antibodies were incubated for 1 hour at room temperature. Details of the antibodies used in this study are shown in Tables S3 and S4.

**Periodic acid-Schiff staining**

PAS staining was carried out using a kit (Sigma-Aldrich 395B-1KT) according to the manufacturer’s instructions. Diastase digestion was subsequently performed to confirm that positive staining was due to the presence of glycogen.

**ALBUMIN and α1-ANTITRYPSIN secretion assay**

The secretion of ALBUMIN and α1-ANTITRYPSIN (A1AT) proteins was determined by commercially available ELISA quantitation kits (Bethyl for ALBUMIN and Genway for A1AT). The values were normalized to the protein content per well.

**Indocyanine green (ICG) uptake assay**

After washing with PBS, the cells were incubated at 37 °C for 1 hour with ICG (Sigma) solution at a final concentration of 1 mg/ml. Then, after washing three times with PBS, the dishes were refilled with culture medium. The cellular uptake of ICG was examined by light microscopy.

**Oil red O staining**

To detect lipids, the differentiated cells were fixed with 4% PFA/PBS and incubated with oil red O (Sigma-Aldrich) for 1 hour. Then, the cells were washed and analyzed by light microscopy.

**Induction of CYP enzymes**

HepG2 cells and the cells differentiated from hiPSCs were treated with rifampicin, omeprazole and phenobarbital for 48 hours. Total mRNA was extracted from the cells. The expression levels of *CYP3A4*, *CYP1A2* and *CYP2B6* mRNA were analyzed using qRT-PCR. The values were normalized to the expression levels of *GAPDH* mRNA. Relevant figures present the gene expression levels relative to levels in cells without drug treatment. The induction of CYP450 enzymes (CYP1A2, CYP3A4 and CYP2B6) was assessed using the pGlo kit (Promega) according to the manufacturer’s instruction. The measured values were normalized to the number of ALBUMIN(+) cells.

**Whole genome sequencing**

Genomic DNA was sequenced with 100-bp paired end on BGISEQ-500 platform (Beijing Genome Institute, Shenzhen, Guangdong, China). The data processing was conducted as described before^2^.

Additional references

1. Mae, S. et al. Monitoring and robust induction of nephrogenic intermediate mesoderm from human pluripotent stem cells. *Nature communications* **4**, 1367 (2013)

2. Mandai, M. et al. Autologous Induced Stem-Cell-Derived Retinal Cells for Macular Degeneration. *N Engl J Med* **376**, 1038 (2017)
